# Probing the ecological and evolutionary history of a thermophilic cyanobacterial population via statistical properties of its microdiversity

**DOI:** 10.1371/journal.pone.0205396

**Published:** 2018-11-14

**Authors:** Michael J. Rosen, Michelle Davison, Daniel S. Fisher, Devaki Bhaya

**Affiliations:** 1 Applied Physics Department, Stanford University, Stanford, CA, United States of America; 2 Department of Plant Biology, Carnegie Institution for Science, Stanford, CA, United States of America; National Cheng Kung University, TAIWAN

## Abstract

Despite extensive DNA sequencing data derived from natural microbial communities, it remains a major challenge to identify the key evolutionary and ecological forces that shape microbial populations. We have focused on the extensive microdiversity of the cyanobacterium *Synechococcus* sp., which is a dominant member of the dense phototrophic biofilms in the hot springs of Yellowstone National Park. From deep amplicon sequencing of many loci and statistical analyses of these data, we showed previously that the population has undergone an unexpectedly high degree of homologous recombination, unlinking synonymous SNP-pair correlations even on intragenic length scales. Here, we analyze the genic amino acid diversity, which provides new evidence of selection and insights into the evolutionary history of the population. Surprisingly, some features of the data, including the spectrum of distances between genic-alleles, appear consistent with primarily asexual neutral drift. Yet the non-synonymous site frequency spectrum has too large an excess of low-frequency polymorphisms to result from negative selection on deleterious mutations given the distribution of coalescent times that we infer. And our previous analyses showed that the population is not asexual. Taken together, these apparently contradictory data suggest that selection, epistasis, and hitchhiking all play essential roles in generating and stabilizing the diversity. We discuss these as well as potential roles of ecological niches at genomic and genic levels. From quantitative properties of the diversity and comparative genomic data, we infer aspects of the history and inter-spring dispersal of the meta-population since it was established in the Yellowstone Caldera. Our investigations illustrate the need for combining multiple types of sequencing data and quantitative statistical analyses to develop an understanding of microdiversity in natural microbial populations.

## Introduction

Large-scale genetic surveys of microbes in complex environments (e.g. the human microbiome [1], oceans [2], and soil [3]) continue to uncover a great deal of diversity at the species level and above. This is not, *a priori*, very surprising. Spatial and temporal heterogeneities in such environments provide (together with complex chemical interactions) myriad opportunities for ecological specialization. Other sequencing efforts have focused on microbial diversity among coexisting individuals within a single species. Perhaps more surprisingly, these too tend to uncover rich, fine-scale genetic diversity or *microdiversity* (e.g. in microbiota of the human tongue [4], marine *Prochlorococcus* [5, 6] and *Vibrio* [7], and the thermophilic archaeon *Sulfolobus islandicus* [8]). Such diversity may be the product of ecological specialization to a multitude of finer scale niches, i.e. *ecotypes* [9]. Alternatively, it may primarily be the result of the evolutionary dynamics of mutation, selection, recombination, and drift acting on populations occupying the same or overlapping niches. Determining what mixture of ecological and evolutionary forces shapes microbial microdiversity in any particular context is a major challenge.

Even without ecological factors, the consequences of complex evolutionary scenarios for diversity statistics are not well understood. Many studies, therefore, compare some aspects of observed diversity to simpler models (e.g. neutral drift and recombination only, [10, 11]) and fit the parameters of these models to data [12, 13]. Such parameters can then be compared across multiple data sets to look for trends [14]. But it is often difficult to know whether the underlying dynamics are well represented by the assumptions or such analyses just represent convenient fits to particular aspects of the data.

To avoid this difficulty, we take a more naive approach, drawing on multiple forms of sequence data (whole genomes, Sanger shotgun metagenome data, and deep amplicon data) and characterizing multiple statistical properties. Rather than inferring parameters of a particular model, our approach is to study ways in which simple null models appear to succeed, when and how they contradict one another, the reasonableness of inferred quantitative parameters, and which features of the data cannot be accounted for by any existing theory, with the hope that together these will suggest scenarios for the evolutionary and ecological history of the population.

We focused on diversity within unicellular *Synechococcus* sp. communities residing in the microbial mats found in the effluent channels of Octopus Spring (OS) and Mushroom Spring (MS) in Yellowstone National Park (YNP), one of the best-studied populations of thermophilic cyanobacteria [15]. These organisms are predominant from 50–70°C, and different *Synechococcus* clusters of 16S rRNA sequences have been associated with specific temperature ranges [16]. A comparison of the genomes of two *Synechococcus* isolates (*Syn* OS-A, with optimal growth range 58–65°C and *Syn* OS-B′, with optimal growth range 51–61°C), reveals an unexpected level of large-scale genomic rearrangements [17]. This was further corroborated by comparison to metagenomic datasets acquired from the same sites [17]. The presence of substantial microdiversity was noticeable even at the low read depths (∼ 5–10×) of these metagenomes [17].

Recently, we analyzed a deep amplicon sequence data set of 90 genomic loci from this population that included both coding and non-coding regions of the genomes, as well as part of the 16S rRNA gene [18]. We showed that roughly 90% of the cyanobacterial population in our local sample is in the same conventionally defined OTU (97% minimum sequence similarity of 16S rRNA), while in the genomic loci there is a great deal of diversity loosely characterized as a “main cloud” presumably containing this 90% of the population, with the rest being outliers a spectrum of distances away. Statistical properties of this diversity, particularly the joint frequency spectra of pairs of synonymous single nucleotide polymorphisms (SNPs) as well as other measures of linkage, clear recombinant alleles, and comparisons of the genome of *Syn* OS-B′ with the amplicon data, revealed a very high level of homologous recombination within the main cloud (and a considerable amount with the outliers, as well). This, we argued, qualifies the population as “quasi-sexual”.

In that work however, we did not focus on effects of selection in the population beyond concluding that the synonymous polymorphisms bore a signature suggestive of genetic hitchhiking. Here, to find more direct evidence of selection, we performed an extensive characterization of the data placing special emphasis on protein coding diversity, e.g. by comparing the distribution of coalescence times for nucleotide and amino acid sequences as well as differences between synonymous and non-synonymous site-frequency spectra and patterns of linkage.

Since the population in each spring is shaped by the history of mixing between springs, inferences of selection cannot be isolated from the dynamics of the metapopulation occupying the Yellowstone Lower Geyser Basin. We therefore investigated scenarios for the history of this metapopulation using information from the spectrum of synonymous and amino acid divergences between *Syn* OS-A and *Syn* OS-B′ and comparing allele frequencies to metagenome reads from a different spring. The genome comparisons suggest that multiple ancestors seeded the metapopulation; the metagenome, along with evidence directly from the statistics of the alleles in the amplicon data, suggests that the metapopulation is well mixed on the time scale of the coalescence of random members of the population. By combining all our results, we aimed to better understand the extensive diversity of this population in the context of its ecological and evolutionary history.

### Summary of data and results

The datasets we analyzed included two complete genomes (*Syn* OS-A and *Syn* OS-B′ with GenBank accessions CP000239 and CP000240), a shotgun metagenome (SRA accession SRX4057997), and deeply sequenced amplicons (SRA accession SRX4055394). These amplicons, once processed by the *DADA* error correction algorithm [19], resulted in a collection of alleles and corresponding population allele frequencies across 180 400bp loci (Appendix 1). (1) We used the statistics of the non-synonymous, *d*_*n*_, and synonymous, *d*_*s*_, distances as a consistency check on the error correction process, allowing us to infer a low false positive rate but a significant number of low-abundance false negative alleles amongst the putative errors. (2) We also infer an elevated level of indel diversity in intergenic regions that cannot be attributed to sequencing errors.

In the section on **The main cloud and the outliers**, we characterize the structure of the population at the allelic level. We begin with the 16S diversity, (3) finding that 92% of the sampled cyanobacterial population is within two SNPs (∼ 0.5% sequence divergence) of the *Syn* OS-B′ genome, but that alleles similar to *Syn* OS-A and even more diverged cyanobacterial subtypes are also present at low levels. (4) At other genomic loci, we study the distribution of pairwise distances between nucleotide and amino acids alleles, finding remarkably good agreement with an exponential distribution—the theoretical prediction for asexual populations with time-independent rates of coalescence. (5) We characterize the “triangles” formed by *Syn* OS-A, *Syn* OS-B′, and the most abundant amplicon allele at each locus, finding that at most loci *Syn* OS-B′ and the most abundant allele are close, while *Syn* OS-A is far from both. However, we found that that a number of loci break this pattern (e.g. all three sequences are very close, or all three are well-separated). (6) In combination with observations of outlier alleles far from *Syn* OS-A, *Syn* OS-B′, or the most abundant alleles, we interpret these results to suggest that there may be a number of “clouds” of allelic diversity present, with genomes sometimes harboring diversity from several of them. (7) Finally, looking at the nucleotide and amino acid allele frequencies across all loci, we again find a distribution similar to that expected of an asexual population, but rather different than one subject to extensive recombination and drift.

In the section on **Comparisons between synonymous and non-synonymous diversity**, we seek evidence of selection via different statistical properties of synonymous vs. non-synonymous sites. (8) The synonymous and amino acid distances between the homologues of the *Syn* OS-A and *Syn* OS-B′ genomes are found to be in good agreement (for ∼ 90% of the genome) with a single coalescence time and a constant rate of accumulation of divergences (with a slight but significant overdispersion for amino acids). (9) Amongst the amplicon alleles, the site frequency spectrum of synonymous SNPs is close to the neutral drift prediction and has a strong GC bias at fixed sites. (10) The non-synonymous sites, by contrast, have a large excess of low frequency polymorphisms and an asymmetry of high frequency A/T / low frequency G/C for transitions. These results are taken to suggest a GC-biased mutation rate without selection on synonymous sites but strong action of selection at non-synonymous sites. (11) Finally, we compare the relative linkage between synonymous and amino acid SNPs within the main cloud versus with outliers and argue that this imbalance again suggests the action of selection.

In the final section, **Stability and variability across space and time**, we compare the amplicon alleles to the two genomes and the metagenome. (12) Supporting our previous results [18], we find additional evidence in the distribution of pairwise distances that *Syn* OS-B′ is statistically analogous to a random population-frequency-weighted allele selected at each locus and that the same holds for *Syn* OS-A within the *Syn* OS-A-like alleles. (13) Comparing the deep alleles to the metagenome reveals that the observed alleles have frequencies correlated to those found in another spring several years earlier.

### The main cloud and the outliers

#### 16S Diversity

A 438 nt portion (V1-V3) of the 16S rRNA gene was amplified using general bacterial primers (this is short enough that we did not need to truncate reads). From 3288 reads, *DADA* infers 289 alleles. Of these, 162 alleles, collectively accounting for almost half the reads, fall 15 − 30% from either *Syn* OS-A or *Syn* OS-B′, likely comprised of predominantly non-cyanobacterial species, with several matching distant organisms also known to be present in the spring. Nearer to *Syn* OS-B′, there is a relatively smooth decay with distance of the abundance of 16S reads out to ≈ 11% (S1 Fig), which we therefore consider as the scale for potential *Synechococcus*.

This range is similar to the sequence divergence of other thermophilic cyanobacteria found in Octopus Spring (OS) far from the *Syn* OS-B′-like cluster (see Fig 2 in [16]). For example, the cyanobacteria J type has a divergence from the A/B cloud of 16S rRNA sequences of 10 − 11% and is not found in our data, while the even more distant I type (≈ 14.5% from *Syn* OS-A / *Syn* OS-B′) is found, up to a single point indel, with 21 reads. Thermophilic cyanobacteria with 16S sequences in the *Syn* OS-A / *Syn* OS-B′ clade have also been observed in hot-springs in Oregon, but not in several other hotsprings outside North America [20], seeming to suggest that these organisms may not be globally well-mixed even on very long time-scales. However, other thermophilic cyanobacterial sequence clusters > 10% diverged from *Syn* OS-A / *Syn* OS-B′, such as the C1 cluster, are commonly found in Japanese hot springs [20]. For context, typical intraphylum distances between cyanobacteria are ∼ 15% [21].

Within the 11% from *Syn* OS-B′ threshold, we find 62 alleles that cumulatively contain ≈ 55% of the 16S reads. (Note, however, for this and other quantitative estimates, that variations between organisms in 16S copy number or amplification efficiency could introduce biases.) Were we to apply the standard procedure of generating *de novo* OTUs via hierarchical (complete linkage) clustering on these 62 alleles, we would infer 28 or 17 clusters using 3% or 6% thresholds. Our error correction is good enough that most of these are very unlikely to be spurious OTUs resulting from sequence or PCR errors. Indeed, they are relatively well separated: the average minimum nearest-neighbor distance to another cluster is 3.5% or 4.2% using 3% or 6% cutoffs, respectively. And of the 17 clusters formed at a 6% cutoff, only 6 are comprised of a single read, with the rest containing multiple reads and 10 containing multiple alleles.

One 16S sequence has 564 reads and approximately 31% of the *Synechococcus*-like reads: it is identical to that of *Syn* OS-B′ (S1 Fig). In addition, there are 9 variants 1 or 2 SNPs away from this allele, and in the 97% minimum sequence similarity cluster (the typical species-defining bacterial OTU) with *Syn* OS-B′ at the center (i.e. out to 6 SNPs), we find a total of 20 alleles with a combined total frequency of 92% of the *Synechococcus*-like reads and a roughly exponential decay in abundance away from *Syn* OS-B′ with a distance scale of $\approx \frac{1}{4}\%$ (1 SNP).

Near the *Syn* OS-A 16S sequence—which was not observed—we find a single allele with only two associated reads: both reads share 3 identical deletions relative to the *Syn* OS-A genome (two adjacent and one 18bp away) and are therefore likely to be real, while the reads differ by one SNP, consistent with being a PCR error. From this, one would crudely estimate that of the *Synechococcus*-like population in this sample, a fraction ∼ 10^−3^ is *Syn* OS-A-like.

(Although resolving SNP-level 16S diversity via high-throughput sequencing is still limited, diversity at a similar scale has been detected in human-associated microbial populations. From vaginal microbiome communities, 6 *Lactobacillus crispatus* alleles within 1 SNP of the most common type were inferred with abundances ranging from > 95% down to well below 1%, similar to the pattern nearby *Syn* OS-B′ [22]. Similarly, in a longitudinal study of two individuals’ tongue microbiomes, 184 pairs of alleles a single SNP apart were inferred, with as many as 20 falling in the same 3% OTU [23].)

#### Nucleotide, amino acid, and intergenic diversity

Excluding the 16S locus, the 89 loci we amplified contain both coding and intergenic sequence. The criteria employed for locus selection have been given previously [18]. The alleles inferred from these amplicons contain sequence overlapping 181 different coding sequences spread over 268 coding sequence fragments (the two ends of each amplicon sometimes sample two fragments from the same coding sequence). Read depth across these loci varied between 1 and 4048 reads with a mean of 909 and median of 579. *DADA* inferred a range of 1–121 alleles with a mean of 38 and median of 36, as well as the frequency for each allele. Of the non-16S loci, 135 have ≥ 300 reads (with a median of 37 alleles): we will refer to these as the *deeply read loci* and, unless otherwise stated, use only these for subsequent analyses.

The most abundant alleles at these deeply read loci have a median frequency of 23% but a range from ≲ 10% to ≈ 95%. That this is comparable to the frequency of the *Syn* OS-B′ 16S allele (31%, see above) is surprising given the expectation of higher diversity at non-16S loci.

**Fig 1:**
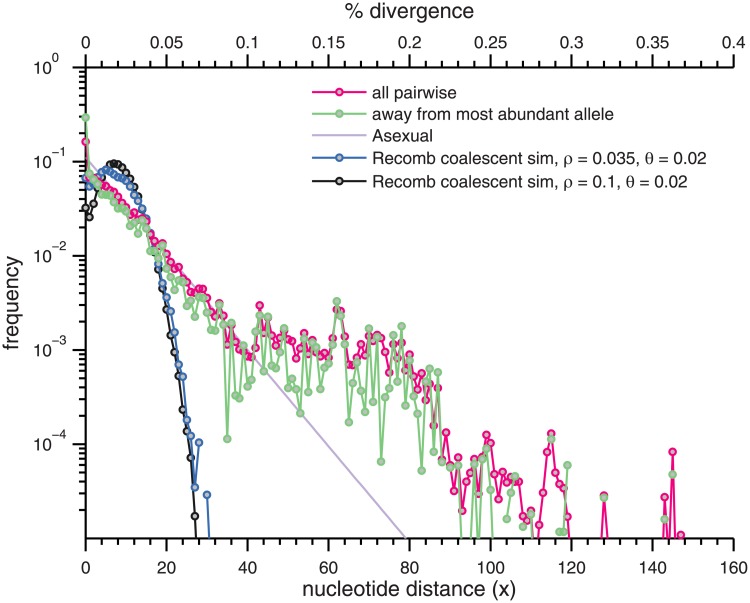
Pairwise distances between nucleotide alleles. The spectrum of pairwise distances *x* (magenta) and distances away from the most abundant allele (green) weighted by allele frequency over the deeply read amplicon loci (with indels excluded). The spectrum for each locus was computed independently, and then averaged together. A geometric distribution with $\bar{x}=8$ (the expectation under asexual, neutral drift) is given in light purple, and the result of coalescent simulation with number of loci and read depths matching those observed, a coalescent-time-scaled per-site mutation rate *θ* = 0.02, and coalescent-time-scaled recombination rates per site of *ρ* = 0.035 and *ρ* = 0.1 are shown in blue and black (see Methods). 44% of the alleles with > 60 substitutions from the most abundant allele and 54% of those with ≥ 80 have only one or two reads.

In Fig 1, the frequency-weighted distribution of distances between all pairs of alleles and also away from the most abundant allele at that locus are shown. A natural distance-scale emerges: the spectra are very similar and strikingly close to exponential with mean heterozygosity *π*_nt_ = 2 × 10^−2^ out to a distance of ≈ 10%, corresponding to the %-scale population-scaled mutation rate per site found in a number of within-species studies [24]: e.g. multilocus sequencing typing (MLST) of *Neisseria meningitidis* [13], MLST of 16 bacterial pathogens and *Candida albicans* [25], whole genome sequencing (WGS) of *Staphylococcus aureus* [26], and WGS of *Streptomyces* soil bacteria [27].

This same distance scale emerged independently in our prior analysis of the dependence of linkage correlations on the diameter of the cloud of sequences included, leading to our loosely defining a “main cloud” of the allelic diversity that includes those alleles ≤ 10% diverged from the most abundant allele at their locus. This turns out to include 92% of alleles and 96% of all reads, comparable to the fraction of 16S reads within the abundant 3% diameter OTU. False negatives were found to have a negligible effects on this distribution (S3 Fig). Likewise, including indels in the distance does not substantially change any qualitative or quantitative features.

Across the 135 deeply read loci, *Syn* OS-B′ falls in the main cloud in 122, is equal to the most abundant allele in 21 (or 24 if indels are neglected), and is found exactly in 54 (or 82 loci if indels are neglected). We studied the distribution of pairwise distances between *Syn* OS-B′ and the alleles of the deeply read loci, both from frequency weighted random alleles and from the most abundant allele (S5 Fig). This revealed the same exponential behavior out to 10% observed in Fig 1. Although with more restricted data, the same was true for *Syn* OS-A in terms of the *Syn* OS-A-like subset (S6 Fig).

The sets of alleles can also be characterized at the amino acid level. Loci vary in their amount of coding sequence, ranging from entirely non-coding to entirely coding. For quantitative comparisons, we kept only the longest coding sequence in each locus (which tends to dominate) and only those loci where this sequence is at least 50 amino acids long and was not annotated as hypothetical. Following this with a screen of having ≥ 300 reads leaves us with 106 coding segments of median length 105 amino acids. Of the full set of nucleotide alleles, these screenings leave ≈ 80% with a range of 5-75 unique amino acid alleles (median 20) per locus.

**Fig 2:**
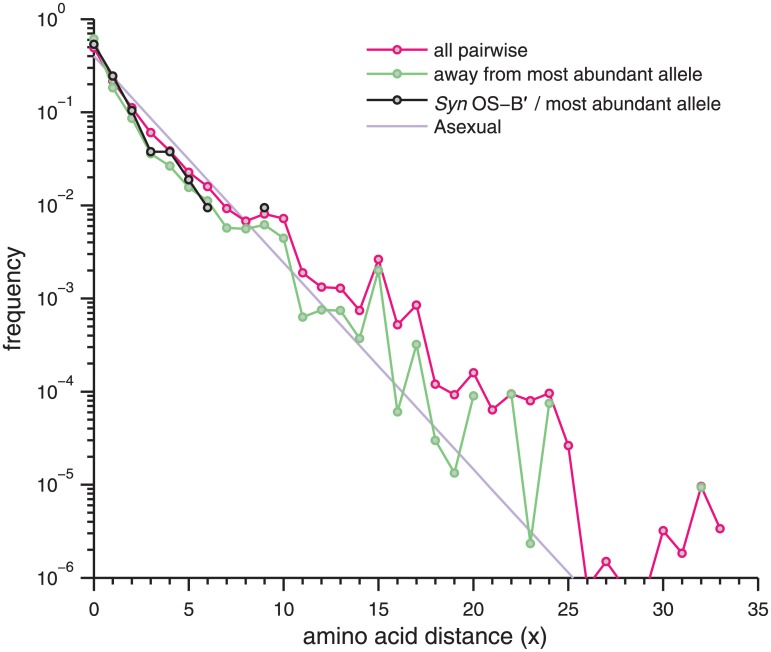
Pairwise distances between amino acid alleles. The spectrum of pairwise distances *x* (magenta) and distances away from the most abundant allele (green) weighted by allele frequency over the amino acid alleles of the 106 deeply read loci with ≥ 50 amino acids (with indels excluded). The spectrum for each locus was computed independently, and then averaged together (although they are not quite comparable, due to the spread in the lengths of the coding sequences). A geometric distribution with $\bar{x}=1.5$ (the expectation under asexual, neutral drift) is given in light purple, and the spectrum of distances from *Syn* OS-B′ to the most abundant is given in black.

In Fig 2, the frequency-weighted distance spectrum between pairs of amino acid alleles (magenta) are shown. Again, a relatively smooth exponential behavior is observed at small divergences, in this case out to ∼ 20 amino acids. The fall-off of the distance spectrum is more rapid for the amino acid than than for nucleotide alleles, with an average distance of 1.5 (light purple), corresponding to a rough per site amino acid heterozygosity of *π*_aa_ = 1.4 × 10^−2^ (with some variation due to the varying lengths of the coding segments). The spectrum of divergences from the most abundant allele (green) is similar so that the main cloud corresponds roughly to alleles at distances ≤ 4 amino acids from the most abundant. The spectrum of distances between *Syn* OS-B′ and the most abundant allele (black) is similar to that of a randomly chosen individual (green), as was the case with the nucleotide spectra.

To compare the nucleotide and amino acid distances, if we assume 3/4 of sites are non-synonymous, the scale of the nucleotide distances per site of 2% from Fig 1 and a typical *d*_*n*_/*d*_*s*_ ≈ 0.12 (see Fig 7 below) translates to *d*_*n*_ ∼ 0.007. Each codon then gets a non-synonymous SNP with a probability $3\times \frac{3}{4}d_{n}\approx 0.016$, very close to what is observed. For the 106 coding loci, the spectrum of synonymous distances also reveals an exponential behavior with an average distance of ∼ 4.5 (S4 Fig), consistent with the typical *d*_*n*_/*d*_*s*_ discussed above. Thus, from the viewpoint of pairwise distances between alleles, amino acids display a pattern consistent with nucleotide, and indeed even synonymous, variation, albeit with a different distance scale that may be accounted for by their larger number of nucleotide sites but typically lower level of diversity. If selection has acted on amino acid diversity differently than on synonymous diversity, the signature of such selection—beyond the conservation of many amino acids—must be sought elsewhere.

Non-coding regions appear to harbor even greater diversity than coding ones. Our nucleotide alleles sample ∼ 14000 non-coding positions (19% of the total) for which the typical substitution diversity per site is somewhat larger than that of the full alleles: the average substitution heterozygosity, for deeply read loci with ≥ 50 intergenic positions, is *π*_intergenic_ = 4.7 × 10^−2^. Indels are especially common in intergenic positions, and including these in the distance raises the heterozygosity to *π*_intergenic_ = 6.6 × 10^−2^. This extra diversity is at least partially associated with a correlation between the amount of intergenic sequence and the fraction of reads that are outside the main cloud. For example, in S7 Fig, we show the relationship between the amount of intergenic (plus hypothetical coding) positions in a locus and the fraction of its reads outside the main cloud.

#### Outlier alleles

We refer to alleles outside the main cloud, i.e. with ≥ 10% nucleotide divergence from the most abundant allele in their loci, as “outliers”. Of the 5467 alleles of deeply read loci, 460 (8.4%) are outliers comprising collectively 3.6% of reads. These alleles mostly have lower frequency than main cloud alleles but they are not dominated by singletons, which comprise just 87 of the outliers. (Note that *DADA* calls singleton reads far from other alleles, as well as some closer to low-abundance alleles, as real.)

To begin to understand the nature of the outliers, we may compare them, and the most abundant alleles, to the two genomes. A significant fraction of the outlier alleles (186) are found ≤ 5% from *Syn* OS-B′ across the 13 loci where *Syn* OS-B′ is outside the main cloud. A similar number appear to be *Syn* OS-A-like. Of the 119 deeply read loci at which *Syn* OS-A is outside the main cloud, in 106 there is some allele < 5% from *Syn* OS-A, with 56 of these having exactly, up to indels, the *Syn* OS-A allele: together, these *Syn* OS-A like alleles comprise 208 of the outlier alleles.

**Fig 3:**
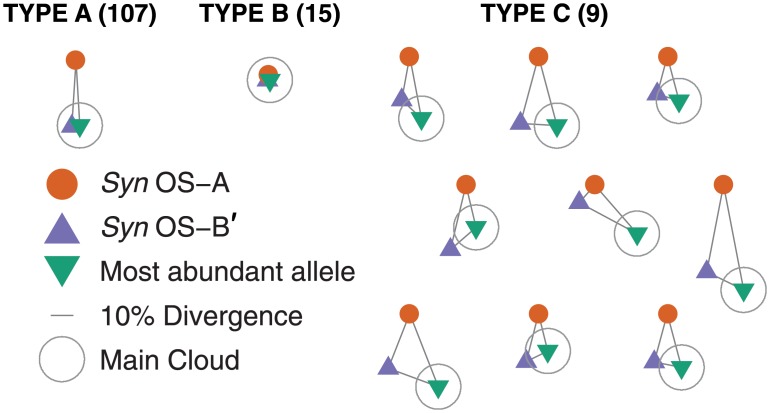
Triangles of *Syn* OS-A / *Syn* OS-B′ / most abundant allele distances. From multiple alignment of the *Syn* OS-A, *Syn* OS-B′, and most abundant allele at each locus, we computed the distances (based on the percentage of columns in the alignment that differ) between each pair of these three alleles. We then formed triangles with edge lengths proportional to these distances for each of the (n = 135) deeply read loci. (Type A) representative of the typical pattern for which *Syn* OS-B′ is inside the main cloud (a 10% radius centered on the most abundant allele, shown by light gray circles) and *Syn* OS-A is outside the main cloud (n = 107, of which 91 have at least one read within 3% of *Syn* OS-A). (Type B) representative of the pattern in which both genomes are within the main cloud (n = 15). (Type C) those for which both genomes are outside the main cloud and also > 10% diverged from each other, with at least one amplicon read found nearby (< 3% divergence) to both genomes (n = 9). Thee were 4 triangles that matched none of these patterns: 1 was like Type C but had no read < 3% from *Syn* OS-A, 2 had *Syn* OS-A inside the main cloud and *Syn* OS-B′ outside it, and 1 had both genomes outside the main cloud but only 3.5% diverged from each other. Allele symbols and a scale bar are given in the lower left.

In Fig 3, the patterns of distances across all loci between the two genomes and the most abundant allele are shown schematically. The most common pattern for the relationships (A) is a long isosceles triangles with the most abundant allele and *Syn* OS-B′ close, and the higher-temperature *Syn* OS-A allele ∼ 10 − 30% diverged so that *Syn* OS-B′ is in the main cloud (*Syn* OS-B′ is seen exactly in 48 of these loci) and *Syn* OS-A outside it. For most of these loci, although the *Syn* OS-A allele is not found, one close to it is. At a small fraction of the loci, *Syn* OS-A is in the main cloud (although exactly present up to indels in just 3), and at 15 loci, both genomes are within the main cloud (B), associated with significantly below-average heterozygosity at those loci ($\bar{\pi }=0.13$ in contrast to $\bar{\pi }=0.20$ for those in (A)), and at 9 of these, all three alleles are ≤ 5% diverged, with several being exactly co-linearly arranged strongly suggesting relatively recent recombination. At an additional 9 loci, all three alleles are > 10% diverged (C), with some number of reads observed near each. These loci are those with the highest overall diversity, having a heterozygosity of $\bar{\pi }=0.49$, more than double that of the typical loci in (A). While some of these have roughly the isosceles shapes found in (A), others are large nearly equilateral triangles, suggesting that additional clouds of diversity (i.e. an *Syn* OS-A-like and *Syn* OS-B′-like) may be present.

In addition to outlying alleles near to the *Syn* OS-A or *Syn* OS-B′ alleles, we find many others far from both of these and from the most abundant allele. Indeed, in the 119 deeply read loci with *Syn* OS-A not in the main cloud, there are 135 nucleotide alleles > 15% diverged from the most abundant, from *Syn* OS-A, and from *Syn* OS-B′. A significant fraction of these are at high abundance and are found at multiple loci (e.g. there are 37 with ≥ 10 reads spread over 8 loci) although the majority are at low abundance (45 singletons, 22 doubletons, 11 tripletons). These numbers are likely to underestimate the diversity of outliers: the use of primers designed to amplify *Syn* OS-A, *Syn* OS-B′, and the moderate number of metagenome reads that aligned well to these genomes has presumably biased against outliers far from all these. A deep non-amplification-biased approach would likely reveal even more outlying diversity.

Despite containing < 4% of reads, the outliers add an additional number of SNPs nearly equal to that of the main cloud. Of these, ≲ 70% are also present in the *Syn* OS-A genome. Many of these extra SNPs are fully linked with one another, suggesting segments in which there has been no homologous recombination between particular outliers and other alleles. These groups of linked SNPs strongly affect linkage statistics and made the estimation of a single recombination rate for the entire sample intrinsically problematic [18].

There are also amino acid alleles very far from both the *Syn* OS-A and *Syn* OS-B′ genomes. We considered the 103 deeply read loci for which *Syn* OS-B′ has a coding sequence exceeding 50 amino acids and *Syn* OS-A has at least 50 amino acids of homologous coding sequence. We aligned each deep allele to the nearer of the two genomes (319 to *Syn* OS-A and 4013 to *Syn* OS-B′), finding just 26 alleles with at least 10 amino acid substitutions. However, if we also include indels in distance, these numbers rise dramatically to 52 closer to *Syn* OS-A and 638 closer to *Syn* OS-B′ having 10+ amino acid distances (indels or substitutions). Because we enforce the coding frame of the genome, and each allele is aligned to by gapping out completely codons with one or more indels (in the nucleotide alignment), and strongly diverged sequences tend to contain many indels, this approach (necessitated by the dominance of indels in coding sequence by errors [S2 Fig] and consequent uncertainty about the coding frame), strongly suppresses the actual amino acid distances to outliers and, presumably, results in underestimating the actual outlying amino acid diversity. And, again, does the use of primers designed to amplify *Syn* OS-A and *Syn* OS-B′.

#### Allele frequencies

A key feature of the diversity, alluded to above but not captured quantitatively by distance spectra, is the spectrum of frequencies of the alleles. This also allows for comparisons between loci, which were all lumped together in the distance spectra.

**Fig 4:**
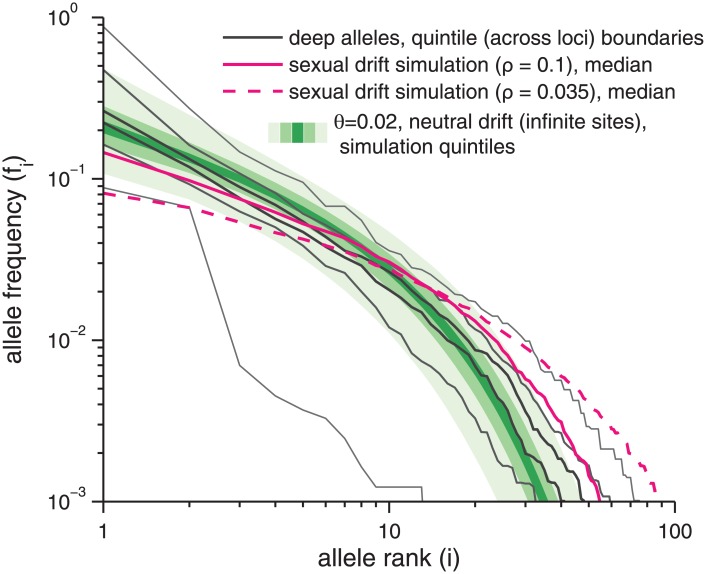
Allele rank-frequency spectrum. The rank frequency spectrum of alleles at the 59 loci with ≥ 1000 reads. At each rank *i*, the black lines designate the quintile boundaries of {*f*_*i*_}, the frequencies of the *i*^*th*^ most abundant alleles, across loci. The shades of green show simulated quintile boundaries over *n* = 1000 replicate simulations for the asexual infinite-sites neutral-drift model with *θ* = 0.02 per site. Magenta shows the median frequency at each rank across a simulated sexual data set with *θ* = 0.02 per site, population-scaled recombination rate per site *ρ* = 0.035 (solid, our earlier best estimate of the main cloud rate [18]) and *ρ* = 0.1 (dashed, an upper bound on the main cloud rate [18]), with coverages identical to those in the true data.

In Fig 4, the allele rank-frequency spectra of the most deeply read loci, the 59 with 1000+ reads each, for which allele frequencies down to 10^−3^ can be studied without being affected by variable depths, are shown. The allele frequencies were rank ordered at each locus, and the vertical ranges at rank *i* delimited by gray lines are quintiles of the frequencies of the *i*^*th*^ most abundant alleles across loci. For example, at rank *i* = 1, these indicate the range of frequencies of the most abundant alleles. Observe that the central 60% of the distributions are quite narrow—a factor of two-to-three range—demonstrating a relatively consistent pattern of allele frequencies for typical loci. No simple form fits the whole rank-frequency spectrum; for example, from the log-log plot shown, it is apparent that no power-law fits well over more than an order of magnitude. In S3 Fig we show that these rank-frequency distributions are negligibly affected by false negatives in *DADA*’s identification of genuine alleles from the raw reads (see Methods). Excluding the outlier alleles also has very little effect (data not shown).

The relatively narrow width (on the log scale) of the center of the rank-frequency spectra masks wide variations in actual frequency spectra across the loci: for example, the distribution of frequencies of all the other alleles are strongly affected by the frequency of the most abundant. Just such behavior occurs in the simplest model of neutral asexual drift in a constant sized population: the quintile boundaries that would be predicted from this model are shown by shades of green. For a population as sexual as previously inferred but neutrally drifting, the probability to observe so many high frequency alleles should be suppressed relative to the asexual model (as seen in the magenta lines in Fig 4), but this is not seen. Instead, we find a number of most abundant alleles (rank 1) with frequencies that exceed the asexual drift range. These alleles dramatically reduce heterozygosity in their loci (S8 Fig), creating a signal of departure from either asexual or sexual drift that is suggestive—in conventional population genetics scenarios for sexual populations—of particularly recent selective sweeps at some loci.

**Fig 5:**
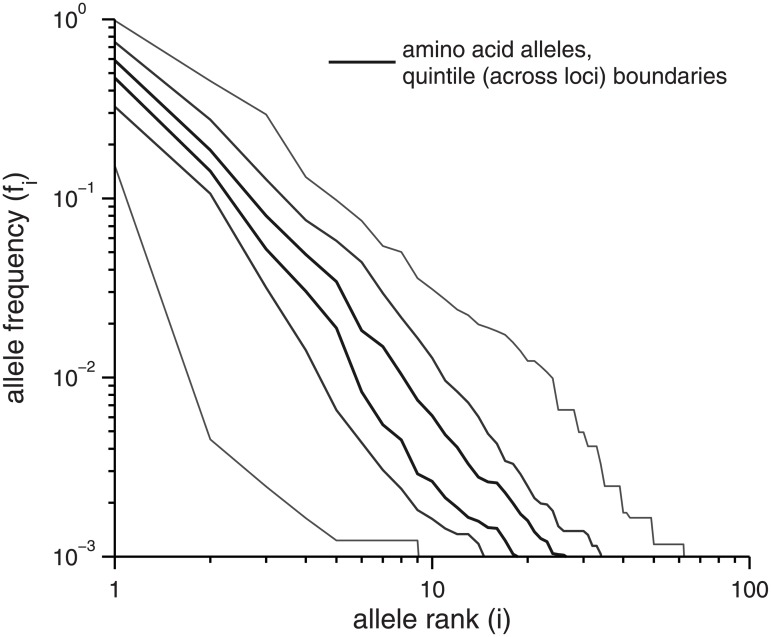
Amino acid alleles rank-frequency spectrum. The rank frequency spectrum of amino acid alleles at the 50 loci with ≥ 1000 reads and longest coding sequence ≥ 50 amino acids. At each rank *i*, the black lines designate the quintile boundaries of {*f*_*i*_}, the frequencies of the *i*^*th*^ most abundant alleles, across loci. Drift null models are not shown due to substantial variations in coding sequence lengths.

In Fig 5, the spectra of frequencies of the amino acid alleles are analyzed similarly and shown. Note that some loci need to be excluded due to too short coding regions (we again chose ≥ 50 aa): thus of the 59 loci with 1000+ reads, only 50 are included here. The amino acid rank-frequency spectra are qualitatively similar to those of the nucleotide alleles, although there are, of course, fewer alleles and each has higher abundance as differences due to synonymous SNPs or in intergenic regions are ignored. The differences in lengths of coding regions makes direct comparisons problematic, but it is evident that the spread across loci of the middle part of the distribution is, as with the full nucleotide alleles, relatively narrow.

### Comparisons between synonymous and non-synonymous diversity

We have considered statistics of the nucleotide and amino acid diversity separately and found that these show qualitatively similar structure. But whether nucleotide differences are synonymous or non-synonymous in coding regions, and thus whether or not they change amino acids, affects how likely it is that, and to what degree, selection acts on them. So we analyzed the similarities, differences, and correlations between the statistics of synonymous and non-synonymous diversity, beginning with *d*_*n*_/*d*_*s*_, the inferred ratio of the rates of non-synonymous to synonymous substitutions, and moving on to comparative studies of the statistics of synonymous and non-synonymous SNPs as well as pairs of SNPs.

#### *d*_*n*_, *d*_*s*_ and inter-locus variation

A traditional test of the extent of selection and its nature is to compare the synonymous to non-synonymous fractional divergence between alleles of different strains or species [28]. A small *d*_*n*_/*d*_*s*_ ratio is generally interpreted to suggest strong negative selection at that locus, while larger ratios may suggest positive selection, diversifying selection, relaxed selection, or the presence of transient deleterious mutations [29]. However, this statistic must be interpreted with care, as it depends not only on selection but on the overall population structure and dynamics [30].

#### *Syn* OS-A to *Syn* OS-B′ divergences

**Fig 6:**
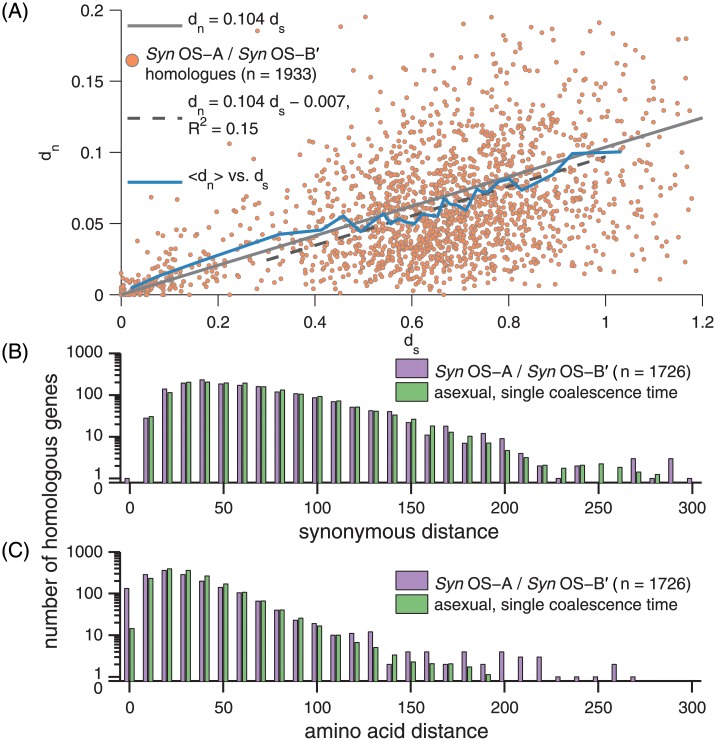
*Syn* OS-A /*Syn* OS-B′ divergence. (A) The spectrum of synonymous, *d*_*s*_, and non-synonymous, *d*_*n*_, distances between *Syn* OS-A and *Syn* OS-B′ for their 1933 homologous genes (defined as reciprocal best BLAST hits with e-values < 1*e* − 100). In blue is a moving average of *d*_*n*_ over windows of size 0.05 in *d*_*s*_. The genome-wide MLE ratio of *d*_*n*_/*d*_*s*_ = 0.104 is shown (gray, from concatenating all loci together, see Methods), as well as the linear least squares fit of *d*_*n*_ = 0.104*d*_*s*_ − 0.007 for the 1726 well-diverged homologues with *d*_*s*_ ≥ 0.3 (dashed black). (B-C) Histograms of the spectrum of synonymous (B) and non-synonymous (C) distances between the well-diverged homologues of *Syn* OS-A and *Syn* OS-B′. (B) Distribution (purple) of the number of conserved four-fold degenerate amino acids (A,G,P,T,V) with at least one synonymous SNP and the maximum likelihood distribution (green) under the assumption of asexual divergence with a single coalescence time, *T*_*c*_ with *θ*_*s*_ = 2*T*_*c*_*μ* = 0.620 the probability for each four-fold degenerate codon to have mutated since the most recent common ancestor of *Syn* OS-A and *Syn* OS-B′. (C) Distribution of the amino acid distances (purple) and the maximum likelihood distribution (green) under the asexual single coalescence time model with *θ*_*n*_ = 0.113. Note the large excess over the null model at small distances.

We first compared *Syn* OS-A and *Syn* OS-B′, for which the full genomes provide many more loci than the amplicon data. In Fig 6A, we show the spectrum of *d*_*n*_ and *d*_*s*_ for 1933 homologous genes of the *Syn* OS-A and *Syn* OS-B′ genomes. Two groups of loci are seen: a small fraction (207/1933) with *d*_*s*_ < 0.3 (equal to an overall divergence of ∼ 10%), corresponding to the 15/135 *Syn* OS-A alleles in the main cloud of the amplicon data, and a large majority of loci with *d*_*s*_ > 0.3 corresponding to *Syn* OS-A alleles being outliers. Many of the well-diverged loci have *d*_*s*_ close to saturated (so that more than one neutral mutation is likely to have occurred at synonymous sites). The genome-wide ratio of *d*_*n*_/*d*_*s*_ ≈ 0.104 (gray line) is similar to typical marine Synechococci ratios: 0.107 or 0.136 depending upon the method [31].

The *d*_*n*_/*d*_*s*_ data in both the low- and high-divergence groups are roughly centered on the genome-wide average, but with a broad spread. When averages of *d*_*n*_/*d*_*s*_ are computed over a sliding window of *d*_*s*_, a slight upturn is seen at low values of *d*_*s*_. As their low synonymous divergence suggests these loci have diverged more recently (e.g. from being recombined from an *Syn* OS-B′-like genome into *Syn* OS-A), their elevated *d*_*n*_/*d*_*s*_ ratio could be the result of deleterious mutations that have yet to be purged—a phenomena that has been evoked previously to explain higher *d*_*n*_/*d*_*s*_ for very closely related genomes [29]. Although we return to this possibility in the discussion, inferring such a trend from comparisons with such a broad spread of *d*_*n*_ vs. *d*_*s*_ is problematic, particularly as the diversity being compared may represent polymorphisms within a population as opposed to divergences between species [30].

The broad distributions of *d*_*n*_ and *d*_*s*_ between *Syn* OS-A and *Syn* OS-B′ genic loci can be analyzed similarly to the deep data. The distance spectra of typical pairs of reads from the deep data, of reads from the most abundant allele, and between the most abundant allele and *Syn* OS-B′, are all similar and close to exponential out to ∼ 10% for nucleotides and ∼ 20% for amino acids, the latter more than the typical distance between *Syn* OS-A and *Syn* OS-B′. In contrast, the spectrum of divergences between *Syn* OS-A and *Syn* OS-B′ has a very different shape.

In Fig 6B the spectrum of synonymous distances across the 1726 loci in the widely diverged group are shown. This fits very well to a Poisson distribution weighted by the numbers of synonymous sites, which would be expected if all of these loci coalesced at the same time in the past. In Fig 6C, the spectrum of amino acid distances is also compared with the Poisson distribution, which fits reasonably well for the majority of loci, but has a large excess of loci with very small distances and some loci with larger distances than expected. Perhaps some conclusions about selection—e.g. that some loci are subject to stronger negative selection—can be drawn from these differences, but they would be rather tenuous. If most of the widely-diverged loci diverged at the same time, there would be no substantial correlation between *d*_*n*_ and *d*_*s*_. Reasonably consistent with this expectation, a linear regression for the widely diverged data shows an *R*^2^ of just 0.15 (Fig 6A, dashed black).

#### Amplicon *d*_*n*_ and *d*_*s*_ divergences

**Fig 7:**
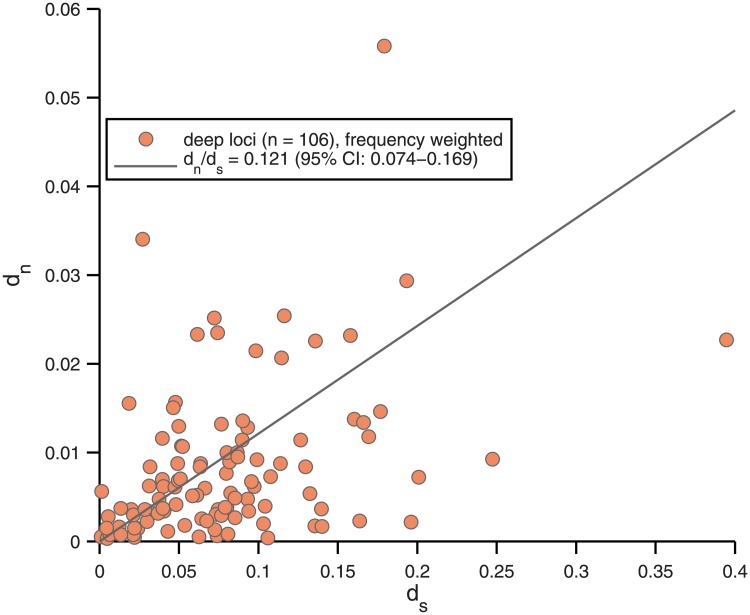
Per-locus allelic *d*_*n*_ and *d*_*s*_. The frequency-weighted averages *d*_*n*_ and *d*_*s*_ between alleles for each of the 106 deeply read loci with coding sequences at least 50 amino acids long. The least-squares fit through 0 is shown, with *d*_*n*_/*d*_*s*_ = 0.121, and a 95% confidence interval on this slope in the legend.

The deep amplicon data also provide information on *d*_*n*_/*d*_*s*_. Although the number of loci are much smaller, there are many alleles at each. We can therefore examine, for each locus, the mean *d*_*n*_ and *d*_*s*_ from randomly sampling pairs of reads. A wide spread is expected because the typical *d*_*n*_ are small: one non-synonymous change typically corresponds to *d*_*n*_ ∼ 0.005 and, over all pairs of reads, the mean number of amino acid changes is 1.76, with a central 95% range of 0-8. The synonymous divergences should have only slightly sharper statistics, with a median number of changes of 5 and a central 95% range of 1-19. Fig 7 shows this spread between loci with a linear fit of *d*_*n*_/*d*_*s*_ = 0.121, having a very poor confidence interval: 95% CI = (0.074–0.169). Again it is hard to conclude much about selection on particular loci from these data. The dependence of *d*_*n*_ on *d*_*s*_ for ∼ 12000 low-temperature OS metagenome reads relative to *Syn* OS-B′ is also consistent with Fig 6, i.e. *d*_*n*_/*d*_*s*_ ≈ 0.1 throughout most of the range, and an upturn to *d*_*n*_/*d*_*s*_ ∼ 0.2 below *d*_*s*_ < 0.1 (S9 Fig).

#### SNP frequency statistics: Drift and selection

The frequency statistics of SNPs provide complementary information to the spectrum of distances between alleles, including additional information on differences between synonymous, non-synonymous, and intergenic sites which may help distinguish between scenarios of the evolutionary history.

There are a total of 400 × 135 = 54000 sites in the deeply read loci. We began with multiple alignments of each locus containing either all deep alleles or just those in the main cloud, in addition to the homologous *Syn* OS-B′ genome sequence. We then extracted from each of these alignments those columns where *Syn* OS-B′ contained a base, so that any insertions in deep alleles are ignored and deletions relative *Syn* OS-B′ result in gaps. 10724 of these columns are non-coding in *Syn* OS-B′. As a representative set of non-synonymous sites, we took all the second positions in the 14266 codons in *Syn* OS-B′: for these, a SNP always alters the amino acid. For a representative set of synonymous sites, we found the most common amino acid at each codon, and if this was any amino acid besides M, W, Stop, or a gap, we recorded the frequency of each nucleotide at the third site amongst the alleles matching this most common amino acid. In addition, we recorded the frequency of the synonymous nucleotide pairs used on the first sites of codons for L, R, or S, the most common amino acids. This resulted in 16924 total synonymous sites.

Of these 16924/14266/10724 synonymous/non-synonymous/intergenic sites, 5260/2190/4264 are polymorphic in the full data and 3016/1360/1688 are polymorphic in the main cloud. As most of the synonymous polymorphisms are dimorphic transitions (SNPs with A & G or C & T), for comparative purposes we focused on these, of which there are in the full data and main cloud respectively 4063/1490/2603 and 2518/1029/1284 such transition-dimorphic sites. Note that within the main cloud, the fraction of polymorphisms that are dimorphic transitions are similar for all three categories of sites with a relatively small enrichment for synonymous sites (83%/76%/76%), while in the full data, this enrichment is somewhat stronger (77%/68%/61%).

**Fig 8:**
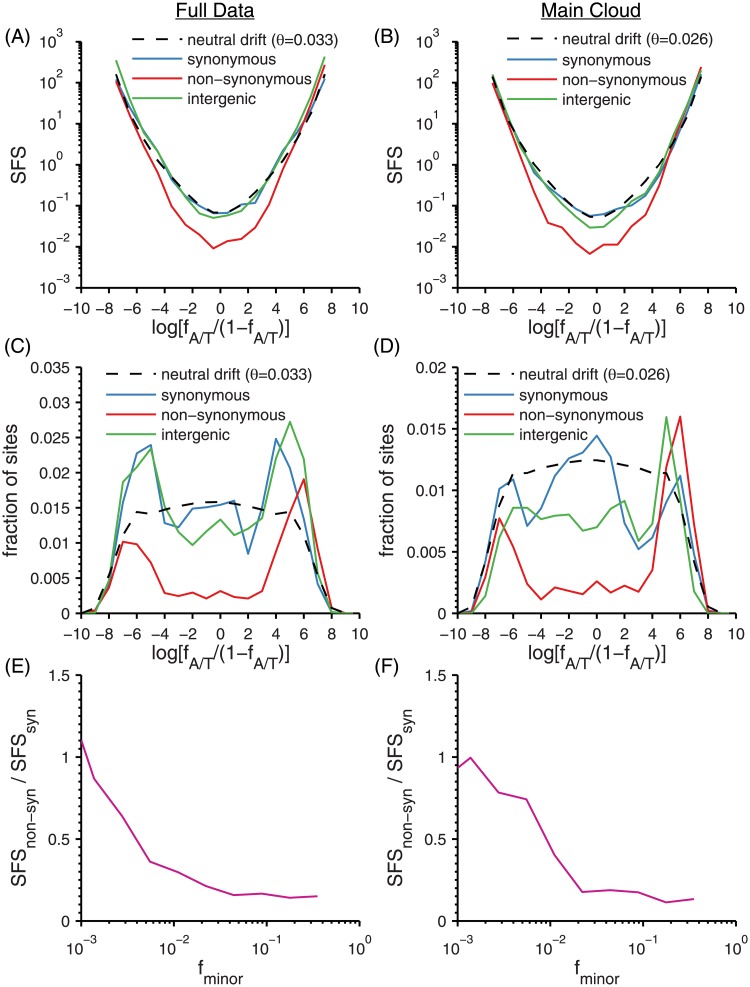
Transition site frequency spectra (SFS). (A-D) Distributions of A/T frequencies *f*_A/T_ of transition SNPs for synonymous (blue), non-synonymous (red), and intergenic (green) polymorphisms, together with equilibrium neutral drift prediction (black dashed) with *θ* inferred from the synonymous heterozygosity. (A,C,E) Full data and (B,D,F) Main cloud. (A,B) SFS vs. the log-transformed variable *ℓ* = log[*f*_A/T_/(1 − *f*_A/T_)]. (C,D) Histogram of *ℓ* without correcting for varying depth. In this ℓ-variable, the neutral drift distribution is roughly flat and equal to *θ*/2 at *ℓ* = 0 (corresponding to *f* = 1/2). The drop-offs at the two ends are due to the varying read depth. (E,F) Ratio of the non-synonymous to synonymous SFSs of transition SNPs vs. logarithm of the minor allele frequency, *f*_minor_ = min(*f*_A/T_, *f*_G/C_). See Methods for details.

The transition dimorphs can be naturally polarized by using *f*_A/T_, the frequency of the A or T nucleotide variant (with *f*_G/C_ = 1 − *f*_A/T_ the frequency of the corresponding G or C variant). In Fig 8A–8D, the site frequency spectra (SFS) of *f*_A/T_ is shown for the synonymous, non-synonymous, and intergenic sites in both the full data (left column) and main cloud (right column). These are plotted in two ways (see Methods): Fig 8A and 8B show the frequency spectrum of *f*_A/T_, demonstrating the slope of ≈ − 1 at extreme frequencies characteristic of the SFS from neutral drift, while Fig 8C and 8D shows the fraction of sites with *f*_A/T_ in logarithmically spaced bins. This latter approach has a flat expectation under neutral drift (modified by the varying read depth) thus providing a clearer view of the asymmetries in the distributions, discussed below. Finally, we show the ratio of the non-synonymous to synonymous site frequency spectra vs. the minor SNP frequencies from $\frac{1}{2}$ down to 10^−3^ (E-F).

#### Synonymous SFS

The synonymous spectra are close to symmetric under interchange of A/T with G/C (Fig 8C and 8D). There are two primary effects that could have given rise to asymmetry. One is selection: the symmetry therefore implies that there is no systematic selection in favor of genomic G/C over A/T, as one might have expected in thermophiles due to the higher stability of GC bonds, but for which evidence has proven elusive in prior studies [32, 33]. The other cause of asymmetry is mutation. If the population was not in mutational equilibrium at synonymous sites—for example due to its ancestors having a lower GC bias—then there would be a mutation-driven probability flux towards GC, resulting in a skew of the distribution towards the AT end. Thus, the data suggest that the population is close to synonymous mutational equilibrium. However, at *fixed* synonymous sites (i.e. those not polymorphic in the sample) there is a large GC bias: 3287:8377 AT:GC (72% GC) in the full data and 4325:9571 (69% GC) in the main cloud. Together with the mutational equilibrium, this strongly suggests that there is a ≈ 2.5 × GC-biased mutation rate (i.e. *μ*(A/T ⇒ G/C) ≈ 2.5*μ*(G/C ⇒ A/T)). This is the opposite bias to that found in studies of microdiversity in pathogens [34, 35]: indeed, it has been argued that in bacteria mutation is universally biased towards AT, and that GC bias on synonymous sites, where present, is due to selection or biased gene conversion rather than mutation. For these thermophilic cyanobacteria, the opposite appears to obtain. We also considered differences in GC bias between the homologues of *Syn* OS-A and *Syn* OS-B′ (Appendix 2) in order to see whether there is evidence that the higher-temperature conditions of *Syn* OS-A had driven an even higher GC bias. We found that for synonymous transitions between the two genomes, *Syn* OS-A had the GC nucleotide variant 58.5% of the time, and 65.1% of the time at A/G ↔ C/T transversions (the other two transversions don’t affect GC content). However, such a pattern could be driven by mutation as well as selection, e.g. if *Syn* OS-A had been subject to a stronger mutational bias.

The shape of the SFS can be compared with the simplest expectation: mutation-drift equilibrium. (The data is easiest to interpret taking out sites with transversion dimorphs or > 2 nucleotide variants: this we do.) In the low mutation rate limit, at all but extremely low frequencies the predicted SFS is approximately $\rho \approx \frac{\theta }{2f(1-f)}$. This form (which is flat in the logarithmic variable *ℓ* = log[*f*/(1 − *f*)]) is shown in Fig 8 with *θ* chosen to match the mean heterozygosity per synonymous site of *π*_*S*_ ≅ 0.033 in the full data and *π*_*S*_ ≅ 0.025 in the main cloud (*π*_*S*_ is equal to the mean genetic distance per synonymous site between pairs of individuals [S4 Fig] and is approximately equal to *θ* in simple mutation-drift equilibrium). Choosing *θ* to give a rough best fit to $\rho \left(f\right)=\frac{\theta }{2f(1-f)}$ results in only ∼ 30% deviations over the three orders of magnitude range of frequencies: thus the neutral drift expectation appears rather good quantitatively. Note that the apparent bimodality in the SFS in Fig 8(C) and 8(D) is due to the limitations of read coverage and the scale used. The read-depth normalized data are consistent with the expectation that the SFS will continue to rise at low frequencies, being roughly similar to the 1/*f* [likewise 1/(1 − *f*)] of the neutral prediction. Fig 8A and 8B, which are corrected for read coverage, confirm, quantitatively, this rise at extreme frequencies.

#### Non-synonymous SFS

Most non-synonymous mutations are not expected to be neutral and thus effects of selection should show up in their SFS. To contrast non-synonymous frequency spectra to the synonymous spectra, we focus on transition polymorphisms of second sites of codons, which are always non-synonymous. The fixed sites were slightly AT biased (6733:5343 AT:GC in the full data and 7116:5751 in the main cloud). The balancing of strong GC bias on synonymous sites with weak AT bias on the (overall) more common non-synonymous sites, overall yields a slightly GC biased genome. The pattern of a relatively strong synonymous GC bias and a weak second-site AT bias is consistent with broad surveys of bacteria [36].

The shape of the spectrum of non-synonymous SNP frequencies in Fig 8C and 8D differs strongly from the synonymous distributions, most obviously by falling well below the neutral drift expectation at intermediate frequencies before rising rapidly at frequencies < 1%. We compare the non-synonymous minor SNP frequencies directly to the synonymous ones by taking the ratio of their SFS (Fig 8E and 8F), finding that non-synonymous SNPs occur with about 1/4 the rate of synonymous ones at mid-frequencies (∼ 1 − 50%), but reach roughly the same rate by frequencies of 10^−3^, the lower limit set by our read depth. S1 Table describes in detail the nature of the non-synonymous SNPs found at extreme frequencies. In short, relative to synonymous SNPs, non-synonymous SNPs tend to be at *low frequency*, isolated to *individual alleles*, and *not found together on the same alleles* (i.e. anti-linked; see also next subsection). This description holds for both the main cloud and even the full data, where the inclusion of the outliers reduces these features only modestly. Considering instead all amino acid dimorphs (rather than only second-site transitions) also preserves the properties of the non-synonymous SNPs, with the main difference being a smaller fraction found on an allele with no other amino acid dimorph due to the much larger space of possible polymorphisms.

In contrast to the almost symmetric synonymous spectrum, the non-synonymous spectrum shows a strong asymmetry in the main cloud with a much larger peak at sites that are mostly AT with a small fraction of GC (Fig 8D). A natural interpretation for the low frequency peaks and asymmetry is that a fraction ∼ 3/4 of non-synonymous second sites are weakly deleterious with the GC bias of mutations making more of these deleterious mutations away from the AT sites: the ratio of the peak heights is roughly consistent with the expectation from the ratio of mutation rates inferred from the synonymous sites. In addition, it would appear that a fraction ∼ 1/4 of non-synonymous second sites are effectively neutral, accounting for the flat region from ∼ 1% to ∼ 99% in the SFS, Fig 8C and 8D. As we shall discuss, quantitative considerations imply that this simple scenario cannot be right.

#### Intergenic SFS

The intergenic spectra bear similarities to both the synonymous and non-synonymous. In the full data, they look similar to the synonymous, being roughly symmetric and having an excess (relative to drift) of SNPs near *f* = 1 − 2% (Fig 8C). In the main cloud, an asymmetry similar to the non-synonymous is observed (Fig 8D), again suggestive of weakly deleterious intergenic SNPs, with the higher plateau for mid-frequencies suggesting a few-times-as-large a fraction of neutral sites.

#### SNP pairs and evidence for selection?

In [18], we showed that the statistics of frequencies of pairs of polymorphic sites can be particularly informative, focusing there on synonymous pairs. To explore the effects of selection, we here contrast non-synonymous and synonymous pairs. Specifically, we analyze the statistics of pairs of dimorphic SNPs *A*/*a* and *B*/*b* with minor allele frequencies *f*_*a*_ and *f*_*b*_. These have four possible haplotypes: *AB*, *Ab*, *aB*, and *ab* with frequencies *f*_*AB*_, *f*_*Ab*_, *f*_*aB*_, and *f*_*ab*_. Studying the joint distribution of these allele frequencies across pairs of sites and especially the dependences on the separation, *x*, between the pair of sites, can reveal much more about the evolutionary processes than single site statistics.

Linkage statistics, which measure the deviations from independence of the haplotype frequencies, *D* = *f*_*ab*_ − *f*_*a*_ *f*_*b*_, give information about recombination: at large *x* in a sexual population, *D* is very small. The *x*−dependence of $r^{2}=\frac{D^{2}}{f_{A}f_{a}f_{B}f_{b}}$ for synonymous pairs was used in [18] to estimate the recombination rate. This and other features of the haplotype correlations show strong dependence on *x* already at tens-of-bp separation in the main cloud and at separations longer, but still substantially less than the 400 bp alleles, even in the full data.

To explore the possible effect of selection by comparing synonymous and amino acid polymorphisms pairs, we face the difficulty that the two have very different single site frequency statistics, and consequently, very different joint frequency statistics (i.e. *P*(*f*_*a*_, *f*_*b*_)). To account for this, we downsampled randomly 1000 times from the synonymous SNP pairs in a biased fashion so as to obtain the same joint frequency spectrum, *P*(*f*_*a*_, *f*_*b*_), as the amino acid SNPs, but without any bias on their conditional pair statistics *P*(*f*_*ab*_, |*f*_*a*_, *f*_*b*_). Thus, this process should yield identical linkage statistics to what would be found for subsets of synonymous sites that collectively match the amino-acid site-frequency statistics. In addition, to prevent the main cloud diversity from “washing out” the signal of the outliers, we removed from the full data pairs of sites if either site was already polymorphic in the main cloud.

Even with this, the pair statistics differ—not surprisingly. But some aspects of how they differ are telling. In particular, the linkage is subtly different between the full data and within the main cloud, as captured by comparing pairs of SNPs both present in the main cloud to pairs of SNPs for which each SNP is present only once the outliers are included. The synonymous pairs of SNPs are substantially more linked within the main cloud, while in the full data the amino acid pairs are comparably linked to the synonymous pairs. This is also seen for the conventional, *r*^2^, linkage statistic (S10 Fig).

**Fig 9:**
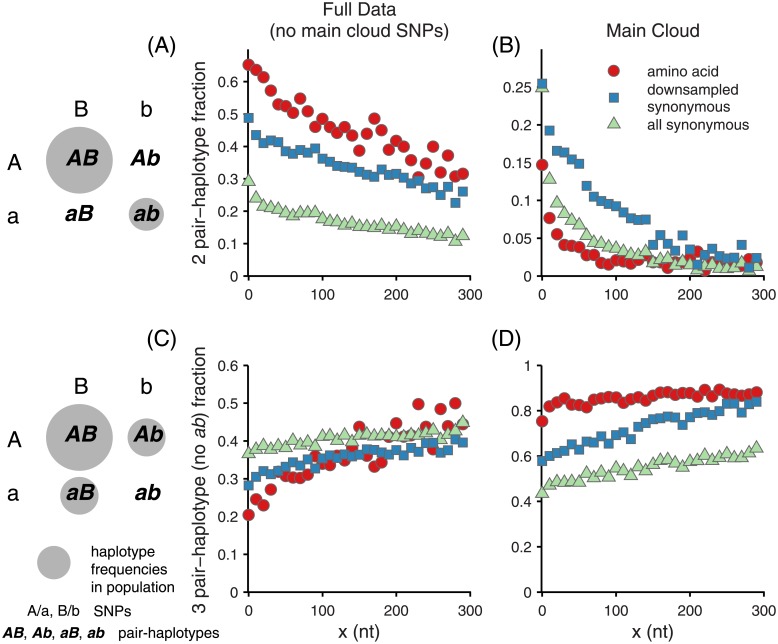
Distance-dependent pair-haplotype probabilities. The probability to observe, from pairs of polymorphisms with nucleotide separation *x*, fully linked pair-haplotypes: (A) full data and (B) main cloud; or triples of pair-haplotypes with *ab* absent: (C) full data and (D) main cloud. Amino acid polymorphisms are shown by red circles, synonymous SNPs by green triangles, and synonymous SNPs downsampled to match the amino acid pair frequency statistics by blue squares. (These data include all first and third site synonymous SNPs). The full data shown in (A) and (C) excludes pairs of sites if either site was polymorphic within the main cloud: e.g., and most frequently, only the AB pair-haplotype is found in the main cloud.

The dominant distinction between synonymous and amino acid linkage is the difference between the fraction of fully linked pairs: *AB*/*ab* for which the other two haplotypes are not observed. Within the main cloud, 10% of down-sampled synonymous pairs were fully linked vs. 3% of non-synonymous pairs, while in the full data, the numbers were very similar, with 36% of synonymous and 49% of non-synonymous, fully linked. Fig 9 shows that these differences represent systematic trends that vary smoothly with the separation of the SNPs: they are not statistical artifacts. The frequencies of the pair haplotype combinations with *ab* absent, the most common, show the opposite pattern to the fully linked combination.

The observed paucity of fully linked pairs in the main cloud could be consistent with the hypothesis that the non-synonymous variation within the main cloud is dominated by weakly deleterious mutations, for which the probability to observe the doubly-deleterious *ab* mutants is strongly suppressed (roughly by the inverse of the scaled fitness cost). By contrast, the rough parity in number of fully linked amino acid and down-sampled synonymous pairs in the outliers suggests that the amino acid diversity of the outliers relative to the main cloud could be neutral or positively selected. Previously we had found [18], from direct observations of close-to-recombinant alleles, that (successful) recombination appears to occur at a similar rate within and without the main cloud despite more extensive linkage outside the main cloud. This suggested that selection, rather than recombination barriers due to sequence divergence, is the primary driver of the linkage of outliers—a hypothesis that predicts an excess of fully linked amino acid pairs relative to comparable frequency synonymous pairs, as seen in Fig 9A.

### Stability and variability across space and time

By using information from several data sets, comparisons can be made across space (between springs), temperature, and time. The multitude of alleles found in the deep amplicon data and their frequencies provide a basis for comparisons with the *Syn* OS-A and *Syn* OS-B′ genomes as well as the previously sequenced shotgun metagenome data from OS and MS [17] and the small sets of amplicon reads from other temperatures in MS. In order to analyze the four subsets of the metagenome data—the high-temperature and low-temperature components of MS and OS-derived reads—we aligned the deep alleles to the metagenome using BLAST, keeping only hits spanning nearly the entire alleles, 836 reads total. For the high-temperature amplicon reads, to maximize the number we could use, we applied a less stringent filtering method than was done for the deep data, resulting in 7156 total reads from the 55, 60, and 65°C samples that overlap at least 200bp of the 400bp deep alleles (see Methods).

**Fig 10:**
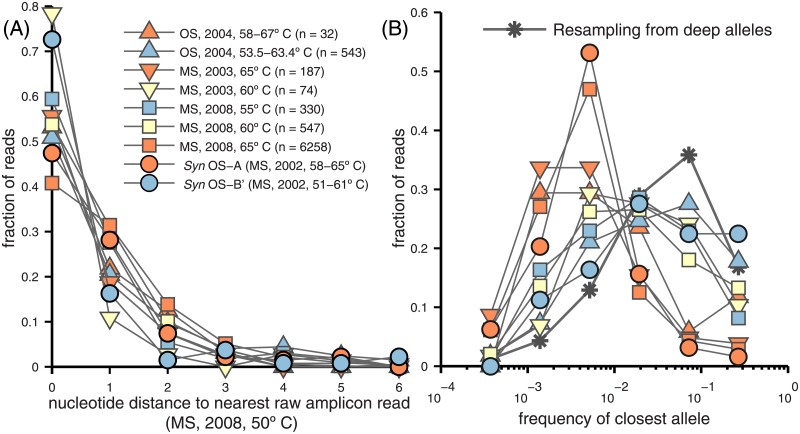
Comparisons between deep amplicon alleles and metagenome data, sequenced genomes, and higher-temperature amplicon data. (A) Distributions of the number of substitutions to the nearest deep read from the high and low-temperature MS metagenome (downward triangle) and OS metagenome (upward triangle); from the two full genomes (circles), and from the high-temperature MS amplicon reads (squares). The temperatures of the data sets and the numbers of reads are given in the legend. Blue, yellow, and red correspond roughly to increasing temperature ranges, although the OS samples experience large fluctuations in temperature so these cannot be strictly compared. (B) Distribution of frequencies of the deep amplicon alleles that were identical to some read from the data sets shown in part (A). If the read from another data set is identical to a raw deep amplicon read but not an identified allele, it is assigned the frequency of the closest allele (from which the amplicon read had been presumed to be an error). The line with stars shows the frequency distribution that would be found if the deep amplicon data sample were resampled, i.e. alleles drawn randomly with probability proportional to their observed frequency. The x coordinates are the left edges of each half-log-sized bin.

In Fig 10, the spectrum of distances of the reads from all the other data sets to the closest amplicon read is shown. We compared with raw amplicon reads rather than the denoised alleles because raw reads that had been judged to have errors because of their proximity to other amplicon reads are in fact likely to be genuine if they exactly match a sequence in another data set: i.e. they were false negatives. (The justification for this assumption is that any *particular* error-containing sequence in the deep amplicon data is not *a priori* expected to be observed.) At least 80% of reads from each data set were within 2 SNPs of some amplicon read and the average distances were in the range 1.1-3.0 SNPs when outliers (those with > 40 SNPs, roughly the radius of the main cloud) were removed.

Where metagenome reads did differ by one or two coding substitutions from the nearest deep read and were similar enough to *Syn* OS-B′ that the genome could be used to establish a clear coding frame (531 of the 836 total metagenome reads), these differences were heavily biased towards being synonymous (21:68 and 29:67 ratios of non-synonymous to synonymous SNPs for reads with one or two coding substitutions), exhibiting (after normalizing for the relative numbers of sites) a *d*_*n*_/*d*_*s*_ ratio at least as small as what we found in Fig 7, suggesting that these differences are predominantly genuine.

Although almost all reads in each of these data sets and the two whole genomes are close to *some* deep allele, one can ask *which* deep allele they are closest to. We previously compared *Syn* OS-B′ with the deeply-read amplicon data [18] by considering, at each locus, the allele most similar to *Syn* OS-B′. We found that the frequencies of these *Syn* OS-B′-like alleles had a very similar spectrum to that of reads drawn randomly and independently with probabilities proportional to the observed allele frequencies in the amplicon data. Here, we similarly analyze the metagenome data and the amplicon data from different temperatures. In Fig 10B, we show that the frequencies of the alleles that reads from different samples are closest to varies greatly between the data sets, the differences correlating strongly with temperature. The sequences from cooler sites in both springs tend to be near to alleles with a spectrum of frequencies characteristic of the deep data, as were *Syn* OS-B′ alleles, also shown. In particular, the 55°C amplicon reads, coming from the same spring and year as the deeply sequenced sample and only 5°C hotter, are, as shown, closest to deep alleles, with a spectrum of frequencies very similar to that of random sampling from the deep data.

In S11 Fig we also consider a more stringent comparison between the low-temperature samples, comparing the frequencies of deep alleles with whether they are found *exactly* in the low-temperature metagenome data. If the metagenome reads could be considered as random draws from the deep data (despite originating from different springs), then the probability for a deep allele to be present in the metagenome would be roughly its frequency. We find that there is indeed a strong such correlation, suggesting persistence in allele frequencies. However only a third as many exact sequence matches are observed as would be expected from this simple model, implying that the correlations are far from perfect.

By contrast to the low-temperature data, the data from warmer sites, e.g. those taken from ∼ 65°C samples, tend to be near low frequency amplicon alleles, similarly to *Syn* OS-A alleles as also shown. As there is a cloud of *Syn* OS-A-like alleles in the deep data with a typical frequency ∼ 1% (S12 Fig) and most of the high-temperature reads are closer to *Syn* OS-A than *Syn* OS-B′ (74%, 86% and 90% of the amplicon, OS metagenome, and MS metagenome reads respectively), substantial similarity between the character of the high-temperature reads, *Syn* OS-A, and the *Syn* OS-A-like amplicon alleles, is not surprising. But the statistical similarities are rather more quantitative. In S6 Fig we compare distance spectra between *Syn* OS-A and the *Syn* OS-A-like cloud of amplicon alleles and find that these are similar to the pairwise distance spectra within the *Syn* OS-A-like cloud. Thus these limited data are consistent with *Syn* OS-A being like a random agglomeration from a different, higher-temperature population, which shares with the lower-temperature population some similar alleles with correlated frequencies.

We also compared the distances between *Syn* OS-B′ and the deep alleles, finding a distribution, S5 Fig, similar to that between pairs of frequency-weighted random alleles, as well as between random alleles and the most abundant allele, Fig 1. Analogous behavior of distance distributions at the protein level were shown in Fig 2. These similarities all support the picture of *Syn* OS-B′ also being like a random agglomeration of the observed population-level diversity, as we found previously in the context of allele frequencies [18].

## Discussion: Scenarios for generating and maintaining diversity

We have observed rich allelic diversity within a sample of a population of thermophilic *Synechococcus* sp., using an error-correcting algorithm and subsequent cross-checks enabling high resolution and quantitative analyses. Almost all of the population has a 16S rRNA sequence within $\frac{1}{2}\%$ of the most abundant, far narrower than a traditional OTU “species” the large number of genomic regions sequenced, the depth of the sampling, and the much larger sequence diversity among the alleles (∼ 8 × that of the 16S) allowed us to study multiple statistical properties of the diversity. At the loci sampled, most alleles lie within a loosely-defined “main-cloud” around *Syn* OS-B′, with varying numbers of outliers. In addition to the primary sample, two fully sequenced genomes and shallow metagenome data from nearby locations and different years provide further information on the persistence and structure of the diversity. The overarching questions are: Why is there so much diversity in a localized population in which the number of potential niches might seem rather limited? And how does this diversity reflect the ecological and evolutionary history of these biofilm populations?

### Time scales of relatedness and diversification

Synonymous SNPs provide information on the time scales over which the diversity was generated. The probability that a randomly chosen pair of individuals differ at a particular synonymous site, the synonymous heterozygosity, *π*_*S*_, is twice the product of the mutation rate, *μ*, and the ratio of the time to their most recent common ancestor—their coalescent time, *T*_*c*_—to the average generation time, *τ*_*g*_. In our data, averaging over all synonymous sites gives a mean synonymous heterozygosity of *π*_*S*_ ≈ 0.04, which is dominated by diversity within the “main cloud” rather than by outliers. Unfortunately, neither generation times nor mutation rates of thermophilic cyanobacteria in natural environments are well characterized. But assuming a generation time in the range of one day (the approximate minimum doubling time of *Syn* OS-B′ in lab cultures [37]) to ten days and a typical bacterial mutation rate of *μ* ∼ 10^−9^ suggests a mean coalescent time in the main cloud of *T*_*M*_ ∼ 10^5^−10^6^ years, with a range of plausible mutation rates giving at least another order of magnitude uncertainty. The Lava Creek eruption that formed the Yellowstone Caldera, where these springs are located, was 6.4 × 10^5^ years ago [38], so a common ancestor of a large fraction of the population on this or a shorter time frame seems reasonable.

The inference of a coalescent time from synonymous diversity assumes that synonymous mutations are selectively neutral. The synonymous site-frequency spectrum (SFS) probes this directly: we find that the synonymous transition SFS (Fig 8C and 8D) is symmetric in A/T ↔ G/C, as expected if neutral. But almost three times as many of the fixed sites are GC rather than AT, suggesting a strong mutational bias towards GC (similar to the GC-bias of TAQ polymerase [39], which was isolated from *Thermus aquatics*, another OS thermophile). Assuming that this bias is specific to thermophiles (other cyanobacteria do not show such a strong bias) would imply that the ancestors must have been thermophilic for at least a few times 1/*μ* generations: i.e. ∼ 10^2^ *T*_*M*_ ∼ 10^7^−10^8^ years. From their synonymous divergence, the separation between *Syn* OS-A and *Syn* OS-B′ occurred a similar time in the past. For 90% of the homologous loci of these two genomes, the distribution of synonymous distances is very well fit by assuming that there is a *single* coalescent time (Fig 6B), that the non-synonymous distances are similar but with a small excess of loci with very low or very high levels of divergence (Fig 6C), and that there are only weak correlations between *d*_*n*_ and *d*_*s*_ (Fig 6A). The remaining 10% of *Syn* OS-A genes are within the main cloud of the allelic diversity. This suggests that these have been acquired from *Syn* OS-B′-like genomes—or vice-versa, as *Syn* OS-B′ contains *Syn* OS-A-like genes—but that very little recombination has occurred between most of the *Syn* OS-A genome and the main cloud since the divergence of *Syn* OS-A and *Syn* OS-B′. Nevertheless, the amplicon data do suggest that *Syn* OS-B′ is not anomalous among the low-temperature organisms sampled in having a substantial *Syn* OS-A-like component of its genome (see Fig 3). The simplest hypothesis—that the presence of *Syn* OS-A-like alleles in the sample is due to a small number of *Syn* OS-A-like genomes—is made improbable by the distribution of frequencies of the *Syn* OS-A-like alleles shown in S12 Fig, which is broad enough that a distinct sub-population is unlikely to be responsible (unless PCR biases vary very strongly from locus to locus).

A plausible scenario is that at least two ancestral types arrived separately in the Yellowstone Caldera. Indeed, the presence of multiple additional 16S genes a comparable distance from *Syn* OS-A and *Syn* OS-B′ as they are from each other, as well as outlier alleles observed for many of the other loci, suggests multiple ancestral organisms. As the primers were only designed to capture diversity previously observed in *Syn* OS-A, *Syn* OS-B′, and the metagenome, they would likely have missed some (or most) alleles that are far from these—perhaps descendants of other original organisms.

In contrast to the evidence for a constant time-to-last-common-ancestor of most of the *Syn* OS-A and *Syn* OS-B′ genomes, the coalescent times within the main cloud appear to be much smaller and more broadly distributed. Averaged over the full alleles, the heterozygosity per site is *π* ≈ 0.02, with roughly half coming from the synonymous sites and the other half from the non-synonymous and intergenic sites combined. With the length of the alleles *L* = 400bp, the average distance between randomly sampled pairs of sequences is *Lπ* ≈ 8 SNPs. Thus, a distance of 1 SNP corresponds roughly to a coalescence time of ∼*T*_*M*_/10 and the 20% diameter of the main cloud to ∼ 10*T*_*M*_. So the range of time scales probed by the diversity we study would appear to be a factor of 10^2^ in the main cloud and somewhat longer including the furthest outliers such as the *Syn* OS-A-like alleles. But there is additional information about even shorter time scales afforded by the high read depth, as discussed below.

Even assuming that the diversification of the main cloud began with the Lava Creek eruption so that *T*_*M*_ < 10^6^ years, a one-SNP distance corresponds to thousands of years (as does one recombination break-point in a locus [18]). Extinction of springs and formation and colonization of new ones, as well as transport of bacteria between springs, very likely occur on much less than million-year time scales and, at least to some extent, on much less than thousand-year time scales, e.g. as earthquakes lead to the formation of new hot springs [40, 41]. Thus, by some measures, the population in the Yellowstone Caldera is likely to be well mixed. In particular, if the ancestry of a selectively neutral segment of the genome of one individual were traced, and selection on other parts of the genome had not strongly altered its history, this lineage would have likely passed through most springs and most locations within them. We will show that this assumption is consistent with some aspects of the diversity data.

### Apparently asexual, neutral evolution within the main cloud

Beyond characteristic time scales, information on the evolutionary dynamics of the population is also provided by the statistics of distances between alleles, the frequencies of alleles, and the frequencies of SNPs. By several measures, these statistics *appear* to be consistent with a simple hypothesis: that the main cloud represents a well-mixed, neutrally-evolving, asexual population—at least on the 400bp genomic length scales probed.

In an asexual population, the distribution of distances between randomly chosen pairs of alleles is a direct reflection of the distribution of coalescent times. If the rate of coalescence of the ancestors of two individuals is independent of time in the past, as it would be in a well-mixed population in steady state, the distribution of distances will be geometric with mean *Lπ* ≅ 8 SNPs. Surprisingly, the data for the main cloud are very well fit by this form out to about 5*Lπ* ≅ 40: Fig 1. However, this does not imply that conventional neutral birth-death drift is the causative process: the geometric distribution is much more general, as it is expected for any asexual population in which the probability of coalescence of the ancestors of a pair of individuals is independent of time in the past [42].

Another indication of neutral drift in a well-mixed asexual population is the distribution of frequencies of the *alleles* in the population, which is predicted to depend only on the *same* parameter as the pairwise distances, *Lπ*. As shown in Fig 4, the observed distribution is also in quite good agreement with neutral asexual predictions, at least for the bulk of the distribution. Yet there is another simple scenario that would result in an allele-frequency spectrum of the same *form*: if the alleles in the local population represent a sampling from a much larger and more diverse meta-population via occasional migration into the spring (or region of the spring), with the relative local frequencies being changed by fluctuations and (local) extinctions—the allelic analogue of Hubbell’s neutral biodiversity theory [43]. But in this case, the allele-diversity parameter would be controlled by the balance between the migration rate and the local fluctuations and be much smaller than the parameter *Lπ* that characterizes the diversity of the meta-population, so that fewer alleles would have been seen and the local population instead dominated by one (or two) high-frequency alleles. Thus, the observation that the allele-frequency spectrum and the distribution of pairwise distances are related by a single parameter supports the well-mixed hypothesis and suggests that the local frequency-changing dynamics are sufficiently slow that they do not dominate changes due to migration.

Focusing on sites rather than alleles, the synonymous SFS also tests the neutral-drift hypothesis. It fits surprisingly well—within ± ∼ 30% over almost three orders of magnitude of SNP frequencies—to the conventional neutral-drift form, *ρ*_0_(*f*) ≈ *π*_*S*_/[2*f*(1 − *f*)] applicable for small *π*_*S*_. It is in the context of the SFS that it is easiest to see why the high read depth provides information on shorter time scales than *T*_*M*_/(*Lπ*). For conventional neutral drift, a new mutant has only a very small probability (inversely proportional to *f*) to rise to a fraction *f* of the population. But if it does, it will typically have taken a time of order *fT*_*M*_. A read depth of *R* probes frequencies down to 1/*R* and therefore time scales as short as *T*_*M*_/*R*. The many loci with *R* > 1000 thus extend the range of times that our data probes from 10^−3^ *T*_*M*_ to 10^1^ *T*_*M*_. If the upper end is the Lava Creek eruption, the lower end would be ∼ 100 yrs. (A caveat: because of the difficulties of separating differences between them from errors, the existence of such very recently separated alleles could, at best, only be inferred statistically from our data.)

The simple expectation for distributions of pairwise allelic-distances and allele-frequencies depend on the assumption that most of the diversity (not only the synonymous) is neutral. For the allele statistics, this can be tested by focusing on amino acids rather than nucleotide sequences. But again the distributions of pairwise distances (Fig 2) and allele frequencies (Fig 5) are quite consistent with the asexual neutral-drift hypothesis, although over only a modest range for the allele frequencies.

By contrast, the non-synonymous SFS is very different than the synonymous SFS. In the main cloud, the ratio of the non-synonymous to synonymous SFS is more than five times as high at frequencies in the 1% range as it is at intermediate frequencies. However, these low-frequency SNPs do not substantially affect the heterozygosity, and thus might not much affect the pairwise-distance spectrum. Furthermore, their primary effect on the allele-frequency spectrum should be on the low-frequency part, for which, in any case, the deviations from the asexual neutral-drift model are substantial. Thus, the simplest scenario is that the intermediate-frequency non-synonymous SNPs are effectively neutral. We return below to possible interpretations of the low-frequency peak in the non-synonymous SFS.

If most of the diversity is effectively neutral, we should be able to understand the time scales of diversification quantitatively. In the standard model of neutral drift caused by birth-death fluctuations, the mean coalescent time in units of the generation time is proportional to the population size: for data or simulations, the inferred value is usually referred to as the “effective population size”, *N*_*e*_. The actual (“census”) population size of the thermophilic *Synechococcus* sp. in the single sample we studied is itself larger than the *N*_*e*_ ∼ 10^7^−10^8^ inferred from the data. The total population at similar temperatures in MS is much larger than in our sample, and that in the whole Yellowstone basin much larger still. Thus, assuming even a moderate amount of mixing, the population size is many orders of magnitude too large for birth-death fluctuations to give rise to the observed diversity statistics, and so fluctuations driven by other more complex processes must dominate. That the range of *N*_*e*_ we find is in the same range as inferred for many bacterial species with global ranges [24] further underscores the disconnect between *N*_*e*_ and census population size, and the implausibility of bacterial population dynamics being driven primarily by conventional drift.

Various non-selective processes can cause much larger fluctuations than stochastic births and deaths, of which the best known are “bottlenecks” lake-dwelling phytoplankton there can be severe periodic population bottlenecks from seasonal changes and the consequent annual succession [44]. But for these thermophilic cyanobacteria the underground-heated water temperature does not vary substantially and such regular bottlenecks are unlikely. However, bottlenecks from extinction of springs and seeding of new springs at different locations after temperature, geophysical, or other changes could play major roles [41]. Smaller scale processes involving large clumps of the mat such as destruction, motion, or transport within and between springs, as well as larger perturbations such as hail storms [45], would also enhance fluctuations as well as mixing up the population. But as long as none of the fluctuation processes change *global*—i.e. Yellowstone-wide—frequencies of SNPs or alleles by large fractions in a single event, these processes together act just like neutral drift and will give rise to the same statistics—generally with non-local sampling of the population, and possibly also with local sampling. In contrast, if the diversity in local populations were neutral and shaped primarily by recent bottlenecks, the synonymous SFS would be different, with much more weight at low frequencies due to mutations and drift that occurred after the bottleneck.

The magnitude of effectively neutral fluctuations that could be caused by non-selective processes is unclear—by many orders of magnitude. But it would be a remarkable coincidence if the neutral coalescent time ended up being the same as the natural historical time for the origin of the main-cloud microdiversity. As we discuss below, selective processes, with effects on the loci studied limited either by recombination or by other factors, are more likely to dominate the apparent coalescent time scales.

### Recombination and inconsistencies with asexual drift

While many statistical properties of the diversity appear consistent with asexual mostly-neutral stochastic drift, our previous study found other properties that strongly suggest the main cloud behaves more like a quasi-sexual than an asexual population [18]. This was based on three lines of evidence: first, direct observation of perfect or close-to-perfect recombinant alleles both within the main cloud and with outliers; second, *Syn* OS-B′ appearing like a (frequency-weighted) random mixture of alleles at the sampled loci; and third, linkage within the main cloud decaying rapidly with genomic distance. The analysis of the present paper focuses on the statistics over short genomic distances and we discuss these first.

A quantitative measure of linkage is the correlation between frequencies of pairs of SNPs. We found that the linkage measure *r*^2^ decays with separation between the SNPs by a factor of four over a scale *ξ* ≃ 100*bp*, both for synonymous and non-synonymous pairs, as shown in S10 Fig.

For loci with length *L* much longer than the linkage scale, *ξ*, neutral dynamics together with recombination would result, roughly, in *L*/*ξ* segments that are approximately independent of each other. The distribution of distances between alleles would then be similar to a convolution of *L*/*ξ* geometric distributions each with mean *πξ*. For the null model of conventional neutral drift with recombination, *ξ* is inversely proportional to the product, *ρ*, of the rate of recombination breakpoints per site, *R*, and the mean coalescent time, *T*_*M*_. The poor fit to theory of *r*^2^ means that *ρ* can only be extracted meaningfully up to a factor of 3 or so (see Fig 2A from [18]). More importantly, the uncertainty in the actual homologous-recombination rate is far larger: estimates from statistical properties of observed genomes are large underestimates of *R* because selection is likely to act against most recombinants. The difficulty is analogous to trying to estimate mutation rates from statistics of *non*-*synonymous* SNPs—unfortunately, there are no neutral markers for recombination analogous to synonymous SNPs. An additional complicating factor is the degree of outcrossing: as recombination is much more likely between physically nearby organisms, the apparent recombination rate will be further reduced from the actual rate.

In spite of the problems with the interpretation of *ρ*, if most of the observed diversity were neutral, one would expect at least rough consistency between various statistical properties. Using a conservative estimate of *ρ* = 0.035 yields a prediction (based on simulation) for the *distribution of pairwise distances* already very different, as shown in Fig 1, than the close-to-geometric distribution observed and predicted for the asexual limit. As this value of *ρ* corresponds to a drop in *r*^2^ by a factor of four over only 300 bp separation (rather than the 100 bp observed), a more appropriate value of *ρ* for comparisons might be *ρ* ≅ 0.1 yielding an even sharper, close-to-Poisson distribution with the same mean distance and thus more striking disagreement with the data (although variations in numbers of close-to-neutral sites from locus to locus should broaden the distribution somewhat). And the even more rapid drop in linkage over 20 bp in the main cloud seen in Fig 9 and S10 Fig suggests that a subset of the population might be exchanging DNA at an even higher rate. The simplest scenario, that successful recombination between more closely related segments occurs at a higher rate because of higher homology, would further suppress the low end of the pairwise-distance distribution. This would exacerbate the discrepancy between the observed spectrum and the neutrally recombining predictions.

While the synonymous SFS is not directly altered by recombination, the *distribution of allele frequencies* is—for example, if recombination occurs between two halves of a pair of alleles, four distinct alleles will result, lowering the frequencies of the original alleles without altering SNP frequencies. Generally, neutral recombination tends to make few alleles with high frequencies, many more with low frequencies and less variability of the distribution from locus to focus. (Indeed, for very high recombination rates, so many combinations of the SNPs would occur that each is likely to be read only once and the number of observed alleles would be equal to the read depth at each locus.) For *ρ* = 0.035, the changes to the distribution of allele frequencies predicted by the drift model with recombination are modest as shown in Fig 4, while for *ρ* ∼ 0.1 the distribution flattens even more at high ranks. Although the discrepancies are less pronounced than for the pairwise-distance spectra, the allele-frequency data also suggest more linkage than inferred from the SNP-pair correlations, *r*^2^.

Taken together, the statistical properties of diversity within the main cloud present a quandary. Some aspects—the synonymous SFS and the pairwise distance and frequency distributions of alleles—look surprisingly similar to asexual, neutral evolution on 400bp scales, while others—particularly the decay of linkage correlations—indicate a high enough rate of recombination that asexual predictions should not be valid. The inconsistency between the high recombination rate inferred from correlations between pairs of SNPs, and the allele-frequency statistics and pairwise-distance spectra that suggest considerably more linkage of multisite properties on 400bp scales, could be indicative of substantial effects of selection on combinations of mutations—i.e. on the alleles—even within the main cloud.

#### Linkage at longer genomic length scales

With recombination break-point rates as high as inferred from the data, most statistical properties should show strong deviations from asexual statistics on both genomic and short scales. Qualitatively, this can be seen by considering the rate at which the ancestors of a single site in an organism’s genome were transferred around between genomes: this is ∼*Rδ* with *δ* the typical length of genomic regions that are homologously recombined. With *δ* in the range of a few kb—a typical estimate for bacteria [46, 47], although possibly not applicable to these cyanobacteria—this suggests that within the main cloud such transfers of the ancestors of a single site occur substantially more often than 10^−1^*T*_*M*_ which is the typical time for a single new SNP to rise to high frequency in one of the 400bp loci. With neutral drift, the effects of these rapid lateral transfers would destroy the linkage almost completely by separations of order *δ*.

At the longest scales, our earlier observation that *Syn* OS-B′ is consistent with being a random frequency-weighted combination of alleles suggests that there is indeed little linkage on scales much greater than the 50kb typical spacing between our sampled loci.

Recently, a collection of BAC clones (with typical sizes near 100kb) from MS samples was used to study diversity on a genomic length-scale (10s of kb), intermediate to the spacings between our adjacent loci and our within-locus diversity [48]. Roughly 500 BACs from 60°C were found to have *Syn* OS-B′-like 16S rRNA alleles (see Fig S3B in [48]). These BACs contain diversity consistent with the percent-scale *π* and *ρ* found in our 50°C sample [18]. On longer scales, paired-end sequencing revealed that only 21% of clones that map to *Syn* OS-B′ appear to be syntenous with it. And an attempt to amplify from these BACs three protein-coding loci found within ∼ 50 kb upstream of the 16S gene in *Syn* OS-B′ (using *Syn* OS-B′-specific primers) was successful for only 72 of the clones. And even within this more-linked subset of the BACs, there is evidence of many recombination events, both recent and historical. Furthermore, the *Syn* OS-B′ genome appears to have a roughly random locus-by-locus association to the rest of the *Syn* OS-B′-like diversity observed (see Fig S10 in [48]), consistent with our prior observations [18]. Thus, it appears that on both gene-scales and genomic-scales, there is indeed very little linkage within the *Syn* OS-B′-like main cloud.

We note that the low degree of synteny observed within the *Syn* OS-B′-like cloud by [48] is less extreme than that between *Syn* OS-B′ and *Syn* OS-A, for which it typically only extends to one to two genes. But the length scales of the synteny normalized by the divergence times are quantitatively similar.

### Selection and site-frequency statistics

The non-synonymous SFS, *ρ*_*N*_(*f*), provides direct evidence for selection—albeit only on single sites. It is clearly different than the synonymous SFS, *ρ*_0_(*f*) (Fig 8), the latter consistent with synonymous transition mutations being neutral. A natural interpretation of the low and high-frequency (i.e. low-frequency of the other nucleotide) peaks in the non-synonymous SFS is that they reflect weakly-deleterious mutations, towards AT or GC, respectively. This seems reasonable qualitatively: as the non-synonymous fixed sites are only weakly AT biased, the inferred mutational bias towards GC would yield three times as many weakly-deleterious low-frequency polymorphisms that are G/C than A/T, consistent with the observed asymmetry of the non-synonymous SFS. But is this interpretation reasonable quantitatively?

Standard population genetics theory (see [49], Eq 39) predicts that for deleterious mutations with selective disadvantage *s*, the SFS is *ρ*_*s*_(*f*) ≈ exp(−*sfT*_*M*_)*ρ*_0_(*f*) (valid as long as *θ* log(1/*sT*_*M*_) ≪ 1). The exponential drop-off of *ρ*_*s*_(*f*) arises because only mutations that arose within times *t* < 1/*s* in the past are likely to survive and these are unlikely to have risen to frequencies higher than *t*/*T*_*M*_. With the sites collectively having a spectrum of selective disadvantages, the ratio *ρ*_*N*_(*f*)/*ρ*_0_(*f*) (for the minor allele frequency, $f<\frac{1}{2}$) gives information on the distribution of *s*. Sites with *s* ≪ 1/*T*_*M*_ are effectively neutral. For small *f*, *ρ*_*N*_(*f*)/*ρ*_0_(*f*) (shown in Fig 8E and 8F) is roughly the fraction of sites with *s* < 1/(*fT*_*M*_). From the observed non-synonymous SFS at mid-range frequencies, this suggests that about 1/4 of the non-synonymous sites are neutral—reasonable. At the opposite extreme are those not observed to be polymorphic: i.e with frequencies less than the lowest observable frequency due to the read-coverage: these represent about 85% of the total (compared with 69% of the synonymous sites: the large fraction corresponding to the small heterozygosity). But at intermediate frequencies, the behavior is puzzling. As *f* goes from 10^−2^ down to 10^−3^, the non-synonymous SFS becomes comparable to the synonymous SFS: this would suggest that a *majority* of the non-neutral sites have *s* ∼ 10^2^/*T*_*M*_ corresponding to *s* ∼ 10^−6^−10^−5^ per generation. This is completely implausible: why should there be such a narrow range of selective disadvantages of a majority of the SNPs? One would expect a broad distribution of the selective disadvantage *s*, from lethal to almost neutral, with only a small fraction lying within any intermediate range of *log*(*s*) that is far from either extreme. (Note that independent of this expectation, there is a more general problem: in mutation-selection-drift equilibrium with *θ* ≪ 1, *ρ*_*N*_(*f*) should generally be less than *ρ*_0_(*f*): this is already almost violated for *f* ∼ 10^−3^.)

The quantitative difficulty with interpreting the non-synonymous SFS is part of a more general problem: to maintain variant alleles (or genomes) at observable frequencies in such an “old” population naively requires that the selective differences among the multiple observed alleles are tiny even within our local sample. The multiple inconsistencies of the mostly-neutral scenario strongly suggest that even the diversity within the main cloud is driven and maintained by more complex selective processes.

### Correlations between springs

In addition to the extensive data from the one MS sample, we have analyzed much more limited data from OS and from other temperatures in MS. Our results on the correlations between springs provide additional evidence against a predominantly neutral scenario for the diversity. We have argued, based on the diversity statistics in the MS sample alone, that the mixing time between springs is shorter than the genetic-divergence and recombination times on a 400bp scale and that much of the local diversity reflects Yellowstone-wide diversity. This scenario predicts that reads similar to OS metagenome reads and alleles similar to *Syn* OS-B′ alleles should both be present in our MS amplicon data: indeed, they are. But only 35% of the OS sample matches any deep MS allele exactly (S11 Fig), far less than would be expected (even accounting for errors—see Methods) if they were sampled from the same genetic pool.

Nevertheless, among the exact matches that do occur, we found evidence of highly correlated allele frequencies: the probability of a random OS metagenome read exactly matching a particular MS allele is roughly proportional to the MS allele’s frequency (S11 Fig). The 65% of the metagenome reads that do not match any deep MS allele typically differ by a SNP or two from the closest deep allele, and are nearly always within a few % of some allele (Fig 10). The closest deep alleles are again found in rough proportion to their frequency. This is consistent with the expectation that an exact vs. close distinction is arbitrary: it will surely depend on the length of the loci.

In a mostly-neutral scenario, the degree of expected correlations depends on the mixing times between the springs. If, on the one hand, mixing occurred on time scales of order *T*_*M*_/10, then mutations could have driven the alleles apart by a SNP or so. But on such time scales, even neutral frequency-changing processes—which should be at least as fast locally as in the whole caldera—would change frequencies that are less than 10% by large relative amounts, de-correlating the allele frequencies between springs. On the other hand, if there were faster mixing but no selection, the allele frequencies would be more correlated than observed. Thus the nature of the correlations in frequencies between springs is inconsistent with a predominantly neutral scenario for the diversity: it suggests, instead, both stabilizing and destabilizing effects of selection.

### Ecology, niches, and sweeps

#### Ecotypes

The opposite extreme to the scenario that most of the diversity is effectively neutral or weakly deleterious is that it reflects a great number of ecologically distinct subtypes: “ecotypes” occupying different “microniches” [9, 48]. These cyanobacteria live along multiple spatial gradients—a horizontal temperature gradient, and sharp vertical light and O_2_ gradients. They are embedded in a polysaccharide matrix so that rapid mixing is prevented, and they likely move, via active and passive processes, slowly compared with their generation time, permitting local adaptation to particular ranges of these gradients, unlike the species considered in Hutchinson’s “paradox of the plankton” [50]. Interactions with other microbes in the mat, which have their own spatial structure, should also produce variations in selection with location. Evidence from their prevalence at different temperatures suggests that there are at least a few identified ecologically distinct clades, in particular *Syn* OS-A-like and *Syn* OS-B′-like [16, 17]. But is it plausible that the “main cloud” diversity around *Syn* OS-B′ found in our single localized sample reflects a collection of a much large number of more-finely-divided ecotypes? And how could such a scenario be inferred in a population with extensive recombination?

If ecological distinctions were driving the diversification of an asexual population, then inevitably it would begin to partition into discrete ecotypes. But how fine the resulting subdivisions would be depends on the extent to which selective sweeps wipe out the diversity of the subpopulations. The software package Ecotype Simulator (ES) has been used to identify putative ecotypes (PEs) within microbial populations [51]. ES models the dynamics as being driven by a rate of drift, a rate of formation of new ecotypes, and a rate of partial selective sweeps that wipe out all the diversity of the ecotype within which they occur, while not affecting other ecotypes. Recombination is assumed to act too slowly to affect the patterns of sequence clustering and is consequently not included in the model [9, 51]. From the sequence diversity of a microbial population at any genomic locus, ES seeks to obtain a best fit set of dynamical model parameters to the pairwise distances between the sampled genomes. These parameters are then used to form clusters consistent with the model, and these clusters are considered putative ecotypes (PEs).

Samples from four temperatures between 60-68°C and up to 12 depths of the *Synechococcus* diversity of MS have been analyzed within this framework [48, 52, 53]. The inferred clusters of the alleles at 7 loci were, via ES, identified as putative ecotypes (PEs), with between 1-24 found for *Syn* OS-B′-like sequences depending on the depth of sequencing and the locus. The spatial distributions of sequences are shown to correlate with PE-cluster membership, implying that ecological effects must be playing an important role, even within the *Syn* OS-B′-like cloud [53]. But a key assumption of the ES analysis is the neglect of recombination, while all the evidence—including from the data that are used to apply the ES algorithm and infer PEs [48]—points to extensive recombination on all genomic scales, as we discuss above. Thus, the conclusion that the PEs represent actual ecotypes in the sense of separate ecologically distinct subpopulations of *organisms* is not justified.

The argument has been made that it is still possible to infer PEs in the presence of recombination, because recombination is too weak in bacteria to prevent the adaptive divergence of ecotypes through the accumulation of niche-specific genes or alleles [48, 54, 55]. This argument begins with the premise that the equilibrium frequency of a niche-specific allele from one ecotype in genomes of a different ecotype is set by the balance between recombination and selection. Quantitatively, this frequency would be *r*/*s* ≪ 1 (with *s* the deleterious selective effect of replacing the niche-specific allele with the foreign one and *r* the recombination rate from one niche to the other), assumed to be too small to prevent divergence except for implausibly small *s* and large *r* [55]. But, unless the majority of the genome participates in niche specificity, approaches like ES must rely primarily on *niche-neutral* loci. And the fate of such loci and whether they ultimately diverge between potential ecotypes does depend strongly on the recombination rate [55]. Yet with the sharp decay in linkage we (as well as [48]) have observed, the scaled mutation and recombination breakpoint rate are similar—*ρ* ∼ *π*—so that there is only of order one mutation per asexually descended genome segment between typical pairs of individuals: recombination thus makes unviable the use of neutral diversity to infer potential ecotypes. A further difficulty in the PE analysis is the reliance on the assumption that alleles which are adaptive in one microniche must be deleterious in another, which need not be the case when recombination is sufficiently high, as we discuss below.

Independent of difficulties inferring ecotypes if they exist, our earlier analyses and the data of [48] discussed above, are not consistent with the hypothesis that the diversity within the *Syn* OS-B′-like cloud reflects primarily-asexual subdivisions of the population. Thus we must search for plausible scenarios that could give rise to and sustain the observed diversity in the presence of extensive recombination.

#### Stabilizing diversity in a quasi-sexual population

Recombination makes the effects of ecological differences far more complex than in asexual populations, with selection acting on genes or combinations of genes with different degrees of environmental specificity, but without necessarily yielding genome-wide partitioning into ecotypes. Even between *Syn* OS-A and *Syn* OS-B′, isolated from very different temperatures, there is evidence for a good deal of recombination, however enough genomic “backbone” might be maintained in each that considering them as distinct ecotypes that occasionally exchange genes seems reasonable. But within the *Syn* OS-B′-like cloud, there is no evidence for such genomic backbones.

Here we discuss some of the factors that could contribute to the *Syn* OS-B′ cloud diversity and outline several scenarios that are not inconsistent with our data, pointing out some of the important caveats.

The simplest scenario is that *selection is primarily at the level of genes* and that this results in a collection of what we call *eco-alleles*, each favored over a different range of environmental conditions, at each locus. However, even if the main cloud is a quasi-sexual population in which selection is not primarily at the genomic level, interactions between genes, with some combinations working better or worse together, surely play major roles.

In a *spatially divided population* with recombination, *epistatic interactions* between alleles at different loci can preserve diversity even in the absence of spatial variation in environmental conditions [56]. The simplest example involves two pairs of alleles, **A**/**a** and **B**/**b**, with haplotypes **AB** and **ab** both fitter than haplotypes **Ab** and **aB**. In an asexual population, the fitter of **AB** or **ab**—say **AB**—would eventually take over the entire population. But if the migration rate is low and the recombination between the loci sufficiently high, then **AB** migrants into a region dominated by **ab** will be broken up too quickly to rise and take over the local population [57]. Thus, in large populations, a stable coexistence between **AB** and **ab**-dominated regions can exist via the equilibrium of recombination, selection, and migration, even though all the alleles will be found at some frequency in each region.

The combination of epistatic interactions between loci, recombination, and spatial structure can in principle stabilize poly-allelic diversity at multiple loci even without any ecological interactions. In this scenario, local samples from different spatial locations with similar conditions would show similar allelic diversity but with different allele frequencies that reflect local balances between migration and selection for the epistatic combinations. Whether or not this is a viable scenario depends on the quantitative interplay of these dynamical processes.

*Ecological interactions and gradients* can of course cause local diversity and thereby induce correlations of allelic frequencies between spatial locations with similar local environments. Most simply, even with the rest of their genomes identical, ecological effects can surely cause coexistence of a few different eco-alleles at some key loci. A potentially much stronger effect is coexistence of organisms with different flexible genes [58, 59]. Variable functional potential via the flexible genome has already been observed in cyanobacterial metagenomes from these thermophilic populations, including phosphonate uptake and utilization [17] as well as iron transport [60]. And in marine plankton, phosphorous [61] and other nutrient utilization abilities [6, 62] conferred by the flexible genome have been shown to be associated with ecological specificity. Each eco-allele at a key locus, or a particular flexible gene or operon (a group of functionally related genes that are contiguous in the genome, expressed together, and can be laterally-transferred as a unit [63]), could have multiple combinations of alleles at other loci that worked well with it. But in an asexual population, the combination that was optimal at the sampled spatial location would drive out the other combinations. In contrast, with recombination and spatial structure, the best of combinations need not eliminate the others (as in the simple example above). A small number of eco-alleles could thus in principle give rise to multiple coexisting alleles at other loci. And between spatially separated samples, the small set of eco-alleles would result in correlations between frequencies of subsets of the alleles at other loci.

While the interplay between epistasis, recombination, and migration is a potential source of extensive diversity and could bootstrap ecological tendencies to coexistence, it is hard to even begin to make any such scenario predictive. At the quantitative level, what are the relevant spatial mixing processes and their magnitudes—both within and between springs? Are the amount of recombination and magnitude of selective effects needed for this scenario to be viable realistic? And how would the primary gradients affect the diversity in a local sample? Statistically, how could this scenario give rise to the observed properties of the diversity and correlations between springs? To begin to address these questions one would need to sequence both populations and multiple individual genomes from several different springs and spatial locations within each [15, 17, 48, 52, 53, 64].

#### Hitchhiking and continual evolution

An alternative scenario to relatively-stable coexistence either at the genomic or genic level, is a population continually stirred up by selection and mixing with recombination strongly affecting the dynamics. In asexual populations, selective sweeps wipe out the diversity unless they are limited by spatial gradients or other differences that make mutations only beneficial in some conditions: i.e. when they primarily sweep through a single microniche (as assumed in the ES model [51]). But when the recombination rate is high enough that a non-driver allele has a substantial chance of being unlinked during the sweep, it can hitchhike to a higher frequency without the diversity at that locus being eliminated [65, 66]. How much recombination is needed depends on the time scale of the sweep. As a sweep that affects frequencies throughout the caldera will be a mixture of local selection and migration both within and between springs, this is a complex process. But some of its effects can be quite general.

When there are multiple sweeps, the statistics of synonymous SNPs are controlled by this “genetic draft” process, quantitatively by the distribution of the probabilities that they become unlinked during the sweeps. We have shown (unpublished work, Jamie Blundell and Daniel Fisher) that with only weak assumptions about the distributions of magnitudes of hitchhiking events, the SFS of neutral SNPs is very similar over a wide range of frequencies to the conventional neutral-drift prediction, and thus the agreement of our data to the latter does not imply that the dynamics is dominated by small changes in frequencies. A key feature of hitchhiking on multiple selective events is that it can yield coalescent times that are much smaller than the population size times the generation time without severe bottlenecking, helping bridge the enormous gap between the “effective” the “census” population sizes [66].

But hitchhiking can leave a signature on the synonymous SNP statistics that drift does not. The consequences of large hitchhiking events on nearby SNPs is to change their frequencies in a correlated manner, in particular, bringing some pairs up from low to high frequency in one hitchhike. This produces an overall excess of frequency-frequency correlations relative to a drift model, which we observed previously [18]. As the recombination times within a locus and the times between typical sweeps that affect the allele frequencies at that locus are not directly related, such hitchhiking will cause changes in pairwise distance spectra and other properties. Thus, even if the variation at a locus were itself completely neutral, dynamics driven solely by drift and recombination would not be the correct null model.

Although, when its effects are averaged over the genome, extensive hitchhiking can result in site and allele-frequency statistics that are similar to conventional drift, large, recent hitchhiking events—e.g. driven by selection on a locus close to one studied—will leave traces in the diversity. Indeed, even a selective sweep that occurred as long as thousands of years ago would be expected to greatly reduce diversity in nearby parts of the genome and show signatures in the data. While we do see evidence of somewhat reduced heterozygosity in a minority of the loci we study (S8 Fig), the effect is not large. This suggests that sweeps are only partial even within the *Syn* OS-B′-like main cloud and thus must be limited in some way. That we do not observe much suppression of heterozygosity in our local sample means that either sweeps must be limited even locally, or there must be sufficient spatial mixing between sweeps to reestablish the local heterozygosity.

The simplest mechanism to limit the effect of sweeps on the diversity in our sample are the vertical gradients through its ∼ 1mm thickness which surely give rise to at least several genic-level niches. Sampling of the psaA photosystem gene at up to 12 depths shows that, indeed, there are gradients in the frequencies of alleles throughout the thickness of the cyanobacterial layer of the mat [53]. Could epistatic interactions with these, averaged through the thickness, give rise to the type of allele and SNP frequency statistics that we observe at other loci? To address this, population sequencing data from several depths is needed.

Both epistatic interactions between genes and local ecological interactions can also limit individual sweeps. A beneficial mutation that arises may sweep through its local population but must migrate and recombine into other suitable backgrounds in order to continue to spread over the full range of conditions for which it confers a benefit. Hence, it may be prevented from completely sweeping by the different genomic backgrounds (as discussed above) that it will encounter. Likewise, differences in the ecology between locations may make the mutation not beneficial in other springs and prevent its spread.

In addition to the direct limitations of individual sweeps, interference between multiple concurrently segregating mutations enhanced by epistatic and ecological interactions are likely to be important—especially given our conclusion from quantitative considerations that even most of the apparently-neutral population dynamics is driven by selection (together with migration) rather than drift. And since mutations and new recombinants can change the local environment and enable new beneficial mutations, evolution can continue even in the absence of external changes. This scenario could be caricatured as a state of spatio-temporal ecological-evolutionary chaos with persistent structure but continual turnover of alleles and combinations of them. Unfortunately, no theory exists for diversity statistics of such a state—even for simple toy models.

### Diversity driven by phages

An alternative hypothesis to conventional explanations of diversity in terms of microniches or neutral diversity is selection for diversity driven by pathogens (or other predation). Phages can certainly play an important role in microbial population dynamics and diversity [67, 68]. And for some marine cyanobacteria, specifically *Prochlorococcus*, podoviruses have been found that can infect some sub-strains but not others [69].

These *Synechococcus* sp. have CRISPR-Cas adaptive immunity systems that can confer some immunity to specific phages [70]. Recently, a viral metagenome was generated from a 60°C portion of OS, and one cluster of viral contigs contained many matches to the CRISPR spacers, suggesting ongoing battles with a viral population specifically predating on these cyanobacteria [71]. This may be producing tightly coupled co-evolutionary “Red Queen” dynamics between the phages and the bacteria and driving selection on short time scales. But to understand how it would affect diversity on much longer time scales requires an understanding of the spatial dynamics, particularly between springs, as well as the co-evolutionary processes.

A possibility that is often proffered for sexual populations [72], is that battles with pathogens can select on diversity *per se*, although there are not general predictions as to how this would be reflected in diversity statistics. Could selection by phages for diversity also be a—or the—primary driving force in these hot springs? To give rise to diversity at most loci, epistatic interactions with variants at the key loci that affect specific phage sensitivity would also be needed—an interesting scenario to explore by looking for correlations between potentially phage specific sequences (e.g. in CRISPRs) and alleles at other loci.

### Future directions

We have carried out extensive statistical analyses of the available datasets on diversity in the Yellowstone hot spring *Synechococcus* populations. However, it is a major theoretical challenge to distinguish between various scenarios that could explain these data. Additional data would be invaluable for guiding theory, and theory should inform which data would be most instructive. Given the decreases in sequencing costs, it would now be possible to study this population without any amplification while achieving a similar depth to our amplicon data (∼ 1000*X*) across the entire genome with a single sequencing run. This would potentially yield much more information and remove amplification biases on allele frequencies—especially important for ensuring that outlier alleles are included in the data. Studying samples from multiple locations and times should provide useful statistical information on correlations and dynamics as well as the connections between allele frequencies and gradients. Multiple full genomes—taken from single cells to avoid culture growth difficulties and biases—would be invaluable, not least to help establish the degree to which the populations are characterized by genic vs genomic diversity. The exceptional value of a mixture of such sequencing strategies has been shown with the abundant planktonic marine cyanobacterium, *Prochlorococcus* [5, 6, 61]. Finally, more extensive viral metagenome data from the associated phage population, as well as deep sequencing of the CRISPRs, could provide insights into one of the potentially strongest pressures and driver of diversity in this thermophilic bacterial-mat population.

## Appendices

### Appendix 1

#### Amplicon data and error correction

This work draws on amplicon sequence data, two complete genomes, and a metagenome. We have described the primary data used (the amplicons) in detail previously [18]. It consists of multiple 400bp sequences from 180 loci throughout the genomes, and was generated by amplification with PCR primers designed to capture diversity within the population—specifically to include all the earlier metagenomic and full genome sequences at these loci. The products were sequenced on the *454* platform. We applied the *DADA* error correction algorithm [19, 22] to the resultant collection of reads—making extensive use of their spectrum of abundances. This produced a collection of putative alleles and corresponding allele frequencies at each locus. A number of non-homologous alleles (∼ 7% of the total, mostly at very low frequency), e.g. matching un-targeted parts of the *Syn* OS-B′ or other species found in the same springs, were filtered out for all subsequent analyses (see Methods). Although the average rate of (mostly PCR produced) SNP errors is roughly 0.63 per read, we used the statistics of *d*_*n*_ and *d*_*s*_ to infer a very low false positive rate from *DADA*. But this indicates that there appears to be a significant false negative rate for singletons—potential alleles with a single read—after error correction (Methods).

*454* data is known to be prone to a high rate of indel errors associated with homopolymers, and *DADA* uses a simple alignment procedure to remove such errors [19]. Thus, as a check on the consistency of this process, we compared rates of indels in coding sequence to intergenic sequence as a function of read position, inferring (1) the presence of a significant level of indel diversity in intergenic sequence (∼ 1.25% per site per read); and (2) the probable persistence at a low level ($∼\frac{1}{8}\%$ per site per read) of indel errors in coding sequence (S2 Fig). Due to this latter observation, whenever coding information is used, alleles are always aligned to *Syn* OS-B′ (or in some cases *Syn* OS-A) and the coding frame of the genome enforced (Methods).

Examining the putative errors declared by *DADA* suggests that some amount of genuine indel diversity is being lost, especially in intergenic regions. For example, (1) 600 sequences declared as errors have a single indel of length ≥ 5, very unlikely to arise from errors [81]; (2) some sequences contain just a point indel but at very high abundance (one contains 130 identical reads); and (3) 13 sequences have an indel of length ≥ 3 with ≥ 10 reads. But because of difficulties in disentangling these from errors, we have not attempted to analyze their effects.

The 400bp loci we study are derived from the ends of 90 amplicons. The *454* sequencing produced reads with a median length of 393 and a central 95% range of 90-505. To get uniform length alleles for comparisons, we ignored those shorter than 400bp (after trimming off the 10bp demultiplexing tag and the 17-27bp primer sequence) and truncated longer ones to this length. Of the 90 amplicons, 27 are shorter than 800bp, and so have some overlap between alleles originating from the two ends with an average overlap length of ∼ 200bp. For these, we do not construct alleles spanning the full amplicon by matching in the overlap region—given the rapid loss of linkage on a 50-100bp length scale, this is not possible to do. We estimate that the effects of sampling twice ∼ 1/2 the length of ≈ 30% of our loci should not be significant.

In addition to the primary sample analyzed, our amplicon data includes smaller numbers of reads from three higher-temperatures, which we make limited use of without error correction (Methods). We also make use of the two prior *Synechococcus* genomes from OS, *Syn* OS-A and *Syn* OS-B′, and a prior metagenome including roughly 2 × 10^5^ ≈ 900bp paired-end Sanger sequenced reads from both MS and OS. Given the lack of any amplification and this sequencing method, the error rate for these data is expected to be much lower than in the amplicon data, and so no attempt was made at error correction. These reads were compared with the *Syn* OS-A and *Syn* OS-B′ genomes to identify *Synechococcus*-like reads (Methods).

### Appendix 2

#### GC bias comparisons of *Syn* OS-A and *Syn* OS-B′

We assessed GC bias at synonymous sites by taking the 1933 pairs of *Syn* OS-A / *Syn* OS-B′ homologues displayed in Fig 6A and pulling out first and third sites in the same way that we do for the SFS. This yielded a total of 750637 synonymous sites. 572053 are conserved between *Syn* OS-A and *Syn* OS-B′, and of these, 431882 (75.5%) are GC. Another 30825 are A/C ↔ T/G transversions, and of these, 27715 (89.9%) are C ↔ G, displaying an even stronger bias. There are also 128125 transitions, and of these, 74883 (58.5%) are GC in *Syn* OS-A and AT in *Syn* OS-B′. Finally, there are 19634 A/G ↔ C/T transversions, of which 12787 (65.1%) are GC in *Syn* OS-A and AT in *Syn* OS-B′. In total, this gives 547267 GC sites (72.9%) in *Syn* OS-A and 519686 GC sites (69.2%) in *Syn* OS-B′ for overall GC biases of 2.69× and 2.25× in the two genomes, sandwiching the value of 2.54× determined from fixed synonymous sites in the full data, but with a moderately higher bias for *Syn* OS-A primarily from the transitions, suggesting the possibility of selection on a subset of sites.

## Materials and methods

### Experimental methods

The generation of the deep, amplicon data was previously described in detail [18]. Briefly, PCR primers were designed to capture the diversity represented by *Syn* OS-A, *Syn* OS-B′, and existing metagenome data [17] across 90 genomic loci. These primers were used to amplify DNA from 50°C, 55°C, 60°C, and 65°C mat samples from MS, longer amplicons were sheared, and all samples were pooled and sequenced on a single *454* Titanium run. Primer sequences were given previously [18].

### The *DADA* error correction algorithm

After sorting the raw reads by which primer (of the 180) they matched and trimming down to a uniform length of 400bp [18], we applied the *DADA* amplicon error correction algorithm to produce a set of putative genotypes with corresponding abundances from the raw reads [19, 22]. Briefly, this algorithm is based on a model of substitution errors in amplicon sequence data with a rate λ_*j*→*i*_ at which base *j* in the sample ends up as base *i* in the sequence of a read. These errors are assumed to be independent and identically distributed across reads and positions. This model is the basis of an algorithm that alternatively estimates the λ_*j*→*i*_ parameters and partitions the reads into blocks that putatively arose from one genotype in the sample.

### Removal of non-homologous outlier alleles

Using BLAST, we identified 515 alleles out of the 6738 inferred by *DADA* that were partially or fully non-homologous to the sequence that had been targeted for amplification in the OS genomes and screened these from all analyses. These included alleles with no hits to OS genomes but good hits to other organisms found in the same mats, un-targeted parts of the OS genomes, and many alleles that appear to have had an insertion sequence introduced into the amplification target. These alleles were concentrated at low read numbers (80% had three or fewer reads) and tended to be extreme outliers (79% fell outside the main cloud). They also tended to cluster in certain loci: 20 loci contained 65% of the screened alleles while 78 loci had no alleles at all to screen out.

### Coalescent methods

*Asexual predictions*: Fig 4 shows the range of frequencies expected for alleles of each rank for a population in drift-mutation equilibrium. This was based on the following procedure. In each of 1000 replicates, for each locus meeting our read depth requirement, we sampled *K* gamma distributed random variables with shape parameter equal to *θL*/*K* with *θ* = 0.02 and *L* = 400 the sequence length for each genomic locus, and a scale parameter of one. *K* was chosen to be 5000. These gamma distributed random variables were then normalized and sorted from largest to smallest approximating a simulated rank-ordered frequency spectra. We then computed quantiles across loci for each such replicate, and averaged the quantiles across replicates.

*Recombinant simulations*: Figs 1 and 4, and S8 Fig show the results of coalescent simulations used to generate predictions for a population in drift-mutation-recombination balance. For each simulation, we began by generating phylogenies using the *ms* program (downloaded May, 2014) [73]. A (recombinant) coalescent phylogeny was created for each locus with a number of leaves given by the read depth of the locus and a population-scaled recombination rate specific to the particular analysis. We then generated a 400nt sequence for each leaf using using *seq-gen* v1.3.3 with *θ* = 0.02 (matching the overall *π* of the data), a transition/transversion ratio of 10 under the Kimura 2-parameter substitution model, and *Syn* OS-B′ as the ancestral sequence [74]. These simulated collections of reads were then analyzed for each figure in the same way as the real data.

### *d*_*n*_ and *d*_*s*_ calculations

*Maximum likelihood method*: The maximum likelihood method used to calculate values of *d*_*n*_ and *d*_*s*_ for Fig 6 and S9 Fig was that of Goldman and Yang [75], which models and accounts for transition/transversion biases as well as base/codon frequency biases, as implemented in MATLAB with default parameters (using the bacterial genetic code). Other methods (those of Nei-Gojobori [76], Li-Wu-Luo, [77], and Pamilo-Bianchi-Li [78]), were tried as well but did not produce qualitatively different results.

*False positive / false negatives estimation method*: A second method of calculating *d*_*n*_ and *d*_*s*_, developed previously in [18], was used for consistency checks on error correction and the estimation of false positive and false negative rates. Briefly, each pair of codons in an alignment of two coding sequences may be identical, synonymous (if they code for the same amino acid but with different nucleotide sequences), or non-synonymous (if they code for different amino acids). The number of synonymous and non-synonymous codon differences is computed for an alignment, and each is normalized by the expected number under the assumption that all differences are due to errors, with the self-consistently inferred nucleotide error rates established by *DADA* during error correction. These give *d*_*s*_ and *d*_*n*_, and no multiple-hit correction is performed on them. Note that in the case of purely false positives, i.e. all differences due to errors, one would find 〈*d*_*n*_〉/〈*d*_*s*_〉 = 1/1 = 1. This leads to ease of use of this method of defining *d*_*N*_ and *d*_*S*_ for estimating false negative and false positive rates. This method tends to produce somewhat larger values of *d*_*n*_/*d*_*s*_ than the maximum likelihood method for comparisons of biological sequences. For example, for the *Syn* OS-A / *Syn* OS-B′ homologues, it gives ≈ 0.15 in contrast to the *d*_*n*_/*d*_*s*_ ≈ 0.10 seen in Fig 6A.

### Site frequency spectra

Fig 8 visualizes the distribution of frequencies of transition dimorphs in several ways.

First, in Fig 8A and 8B, we show the site frequency spectrum (SFS). To obtain this, we counted the number of SNP frequencies *f*_A/T_ that fell within bins of size 1 (with integer edges) in the variable *ℓ* = log[*f*_A/T_/(1 − *f*_A/T_)]. Then, we divided the counts for each bin by the product of the bin width (in *f*_A/T_) and the number of sites with read depth *R* exceeding the inverse of the smaller frequency bin edge. Without conditioning on read depth in this way, alleles with *f*_A/T_ < 1/*R* may appear as singletons and get counted in the bin containing *f*_A/T_ = 1/*R*, and with such a rapidly rising tail of small frequencies, this creates the risk of significantly overestimating the SFS in this bin. This distribution (computed for synonymous, non-synonymous and intergenic sites) is shown along with the expectation from neutral drift.

To obtain the neutral drift expectation we used the theoretical prediction for the neutral SFS, $\rho \left(f\right)=\frac{{\left[f\left(1-f\right)\right]}^{-1+\theta }}{B(\theta ,\theta )}$, a beta distribution with both parameters equal to *θ*. From this we obtained the expected distribution for the number of reads *x* out of a total of *R* reads for a synonymous site (corresponding to the number with the A/T variant):

$$P(x|R,\theta )=\left(\begin{array}{c} R\\ x \end{array}\right)\int_{0}^{1}\rho (f)f^{x}{\left(1-f\right)}^{R-x}df=\left(\begin{array}{c} R\\ x \end{array}\right)\frac{B(x+\theta ,R-x+\theta )}{B(\theta ,\theta )}$$

This beta-binomial distribution was then summed over synonymous sites, using the read depth *R* for each, and histogrammed into the same frequency bins used for the actual data (described above). Finally this was normalized by the number of synonymous sites with sufficient depth in each bin.

In Fig 8C and 8D, we transform each SNP frequency to the *ℓ* variable and plot a normalized histogram of the distribution of these values (with the same binning as in Fig 8A and 8B). There is no conditioning on read depth. This transformation was applied because it makes the expected distribution under neutral drift (to which the data is relatively close) flat in the limit of low heterozygosity, allowing it to be more easily plotted on a linear scale, revealing finer scale features.

Finally, in Fig 8E and 8F, we show the ratio of the non-synonymous to synonymous SFS as a function of minor SNP frequencies. For this plot, binning was done in powers of 2 starting with $\frac{1}{2}$ and going down to ∼ 10^−3^. The two histograms were normalized by their respective total numbers of sites and their ratio taken.

In all six panels, data points were placed in the center (in log-space) of each bin, and connected into lines.

### False negatives: Estimate of rate and random sampling procedure

Several of the distributions in the manuscript could potentially be affected by false negatives: i.e. alleles declared as errors by *DADA* but actually present in the sample. We estimated the numbers of these by assuming that the declared errors are a mixture of actual errors with 〈*d*_*n*_〉/〈*d*_*s*_〉 = 1 and genuine diversity with predominantly much lower 〈*d*_*n*_〉/〈*d*_*s*_〉, as suggested by Figs 6A and 7 and other studies of cyanobacteria [31], using everywhere our internal definitions of *d*_*n*_ and *d*_*s*_.

After filtering out non-homologous outliers and their associated errors and ignoring indels, *DADA* inferred that there were 48893 sequences that contain one or more errors that fell within coding regions. These consisted of (1) singleton reads with ≤ 7 substitution differences in their coding sequence from their putative inferred-real genotype, (2) sequences with 2 − 10 reads and one inferred coding sequence substitution from a more abundant inferred-real allele, and (3) two-read sequences with two coding sequence substitutions (“two-aways”) from a more abundant inferred-real allele.

What follows is a simple procedure we developed for sampling random sets of potential false negative sequences from this collection of putative errors which conform to a prior expectation of 〈*d*_*n*_〉/〈*d*_*s*_〉 for biological sequences while leaving behind a collection of errors with 〈*d*_*n*_〉/〈*d*_*s*_〉 = 1—as expected for true errors. These random sets of sequences were then used to assess the robustness of our analyses to the presence of false negatives alleles.

For each collection of putative error-containing sequences with *n* reads and *d* substitutions in coding sequences relative to their inferred-real alleles, we identified the number of sequences $\left\{s_{i,d}^{\left(n\right)}|i\in 0\ldots d\right\}$ for which *i* out of the *d* errors were synonymous substitutions. We then sought to divide each collection of $s_{i,d}^{\left(n\right)}$ sequences into a number $x_{i,d}^{\left(n\right)}$ sequences that were the result of errors and a number $r_{i,d}^{\left(n\right)}=s_{i,d}^{\left(n\right)}-x_{i,d}^{\left(n\right)}$ that were the result of false negatives.

If coding sequences are much longer than *d* and if all substitutions are due to errors, then the $x_{i,d}^{\left(n\right)}$ should be be distributed (in *i*) proportionally to a binomial with *d* trials and success probability *p*_*S*_, the chance for each error to produce a synonymous mutation. For the set of false negatives we estimated the *d*_*n*_/*d*_*s*_ ratio to be 0.15 based on arguments given previously [18] as well as using a more liberal *d*_*n*_/*d*_*s*_ = 0.30. This latter value is useful, as for such low abundance alleles near to more abundant ones, the biological *d*_*n*_/*d*_*s*_ ratio may be larger than we previously estimated, and false negative alleles may contain a mixture of genuine SNPs and errors, both of which would drive up the *d*_*n*_/*d*_*s*_ expected for genuine alleles.

Enforcing both the above conditions allows us to solve for the all the $r_{i,d}^{\left(n\right)}$. For some of the *n*, *d*, we get a small negative value for *r*_0,*d*_ (those with only non-synonymous changes), which we set to 0. We then drew random sets of $r_{i,d}^{\left(n\right)}$ sequences for each {*n*, *i*, *d*} to represent a random sample of false negatives. For *d*_*n*_/*d*_*s*_ = 0.15, this resulted in 7143 total FNs, while for *d*_*n*_/*d*_*s*_ = 0.30, we obtained 10754 FNs (Table 1).

**Table 1:** False negative estimation.

	putative errors	FNs, *d*_*n*_/*d*_*s*_ = 0.15	FNs, *d*_*n*_/*d*_*s*_ = 0.30
singletons, ≥ 1 CDS errors	42386	6240	9358
multi-read, 1 CDS error	5861	749	1157
doubletons, 2 CDS errors	646	154	239

The number of *DADA*-inferred putative error-containing sequences characterized by number of reads and number of errors in coding sequences (putative errors) with inferred number of false negatives (FNs) for a conservative (*d*_*n*_/*d*_*s*_ = 0.15) and liberal (*d*_*n*_/*d*_*s*_ = 0.30) estimate of the *d*_*n*_/*d*_*s*_ ratio for biological sequences.

These randomly drawn putative false negative alleles were used for some reanalyses. S3 Fig demonstrates that the spectrum of pairwise distances of Fig 1 is unlikely to be much affected by the presence of false negatives. Fig 4, the allele rank-frequency spectrum, was also remade with the inclusion of these randomly drawn false negatives, and no effect at all was seen (not shown).

### Metagenome reads: Recruitment to the *Syn* OS-B′ genome and deep alleles

The metagenome consists of roughly 200,000 reads, about 95% of which exist in mate-pairs, with a median length of 853 nt [17]. We divided these reads into four subsets: high (58 − 67°C) and low (53.5 − 63.4°C) temperature OS reads as well as high (65°C) and low (60°C) temperature MS reads (∼ 6% had tags that did not allow their spring/temperature to be identified). Low-temperature OS reads were the most abundant, comprising roughly half of the total. We then applied the following two simple BLAST-based pipelines for recruiting these reads to the *Syn* OS-B′ genome and the deep alleles. BLAST was chosen for its high sensitivity in detecting alignments of sequences that are well diverged from the reference and because the moderate size of our datasets made its use possible.

*Recruitment to Syn OS-B′*: We used BLASTn v2.2.26 [79] with word size 8 to align metagenome reads to the *Syn* OS-A and *Syn* OS-B′ genomes, finding 58552 reads with at least one hit having an e-value < 10^−10^ (the e-value for a BLAST hit is an estimate of the expected number of hits of equal of better score given the database size and read length). Of these, we focused on the 32787 with their lowest e-value hit to *Syn* OS-B′, 20462 of which had hits that spanned the entire read, with 19658 also having no overlap to an insertion sequence (which are abundant in these organisms and make mapping to the genome difficult in some cases [80]). Of these, 17207 were from the lower-temperature OS samples, and 17060 overlapped at least 10 amino acids of at least one coding sequence in *Syn* OS-B′. Finally, of this set, we kept just the 12194 reads with alignments to *Syn* OS-B′ that contained no gaps. The reason for this strict requirement is that we have some evidence of compensatory indels that shift the coding frame and so cause a large number of changes over a short region. It was this final set for which *d*_*n*_ and *d*_*s*_ relative to *Syn* OS-B′ were calculated in S9 Fig.

*Recruitment to the deep alleles*: In a separate pipeline, we used BLASTn v2.2.26 [79] with word size 8 to align the complete collection of metagenome reads against the complete collection of inferred amplicon alleles (after screening non-homologous outliers) in the deeply read loci. 2755 metagenome reads had a hit to at least one allele with an e-value < 10^−10^. To obtain from these a set of *Synechoccoccus*-derived reads appropriate for comparison with our alleles, we kept only BLAST hits that were at least 390bp long, so that they span nearly the entire deep allele obtaining 32/543 high/low-temperature OS reads and 187/74 high/low-temperature MS reads. These were the reads used in comparisons with the deep alleles in Fig 10 and S11 Fig. This number of reads (836) is consistent with a simple estimate: there were ∼ 5 × 10^4^ *Syn* OS-B′-like reads and our primers sampled ≈ 1.8% of the *Syn* OS-B′ genome (135 loci × 400bp / 3 Mb).

*Differences between metagenome reads and deep alleles*: In S11 Fig, we compared the low-temperature OS reads to the deep alleles, finding that just ∼ 34% were an exact match (up to indels) to an allele. Could this low rate of matching be driven by errors? Even assuming a very high Sanger-sequencing error rate of 10^−4^ gives a 96% chance to be error-free over 400bp. And for the amplicons, even if we assume that *DADA* is consistently assigning the allele to sequences with 1-2 errors and this is creating the problem, we see in Fig 10A that just 51% of OS reads match any raw read. The inferred probability to be error-free (per read, up to indels) from *DADA* is typically ∼ 54%. Therefore, for a *DADA*-inferred allele with *R* associated reads, the probability that not even one is error-free is ≈ exp(−0.54*R*). Thus, an appreciable fraction of alleles with 1-4-reads may contain no error-free read, but given the overall allele-frequency spectrum and the small weight in such low-frequency alleles, errors cannot account for the lack of exact matches of the majority of low-temperature OS reads to deep alleles.

### Higher-temperature amplicon reads

In addition to the 50°C MID tag, ACGAGTGCGT, that was used to pull out the amplicon reads that went to *DADA* and generated our deep alleles [18], mat core samples were also collected in 2008 from 55°C, 60°C, and 65°C samples of MS. DNA was extracted and amplified with the same primers as used on the 50° reads, amplicons were tagged with three additional MID sequences for demultiplexing (ACGCTCGACA, AGACGCACTC, and AGCACTGTAG), and all four samples were pooled and sequenced on the same *454* run. Although the amplicons had been mixed in equal proportions, a much smaller number of reads were obtained matching the three higher-temperature MIDs: of the ≈ 1242000 total reads, 1071339 began with the 50°C MID, while only 1020, 1292, and 3398, respectively, began exactly with one of the three higher-temperature tags.

164877 of the reads that did not begin with a MID tag were at least 30bp long. To examine whether some of these might have been errors away from an expected tag, we aligned the first 30bp to each MID sequence via standard global alignment with ends-free gapping on the right side of the alignment but not the left (so that the expected tail beyond the MID in the 30bp subsequences of the high-temperature amplicons would not be penalized), and each amplicon was recruited to the MID that provided the highest scoring alignment. Low scoring alignments (i.e. from amplicons not particularly close to any of the MIDs) were screened out with an empirical score cutoff, while the remainder were generally concentrated in a few sequences very similar to one of the MIDs. Combining the exact and inexact high-temperature MID-matching reads resulted in 1418/2022/14617 reads. Each of these collections of reads was aligned with BLASTn v2.2.26 [79] with an e-value screen of 10^−50^ (empirically determined to effectively filter out non-homologous sequences) and word size of 7 (the small number of reads made possible a slightly smaller word size than our other alignment pipelines) against the set of the amplicon sequences in *Syn* OS-A and *Syn* OS-B′, the MID and any primer sequences stripped, and only the part overlapping the 400bp deeply read loci kept. Reads were then discarded if shorter than 200bp, resulting in final read counts of 330/548/6278 for 55°C/60°C/65°C.

## Supporting information

S1 Table Comparison of synonymous, non-synonymous, and amino acid numbers of low-frequency polymorphisms. TD = transition dimorph, D = dimorph. < 1% freq means that the minor variant of the polymorphism was at < 1%. “One allele” means the minor variant of the polymorphism was found in just one allele. “Alone” means the polymorphism was not found on an allele with any other < 1%, one allele polymorphisms.
Within the main cloud, 75% of non-synonymous transition dimorphs are at < 1% frequency (compared with just 37% for the synonymous). Of these, 95% have their minor variant present in only one allele (compared with 83% for synonymous). Of this subset, 70% are alone (i.e. on alleles that contribute only one such SNP) compared with 56% for synonymous. No allele contributed > 3 < 1% frequency non-synonymous SNPs (compared with 16 for the synonymous).
In the full data, a similar pattern was observed. 72% of non-synonymous transition dimorphs are at < 1% frequency, 87% of these are found in one allele, and 63% of these are alone. 38 alleles contribute > 3 < 1% frequency non-synonymous SNPs, and 9 is the maximum. So any linkage contributed by low-frequency outlier alleles does not overwhelm the features of the non-synonymous SNPs of the main cloud.
Amino acid polymorphisms are similar: 71% of amino acid dimorphs in the main cloud have minor frequencies < 1%, of which 95% exist in only one allele, but (due to the inclusion of differences other than single second-site non-synonymous SNPs) only 47% of amino acid dimorphs are found alone, and the maximum number on a single allele is 13. In the full data, 70% of amino acid dimorphs are at < 1% frequency, 86% exist in only one allele, and 42% are alone. 35 alleles contribute > 6 < 1% frequency amino acid changes, and 23 is the maximum.
(PDF)

S1 Fig *Synechococcus*-like 16S rRNA alleles. Abundance vs. distance to *Syn* OS-B′ (not including indels) for the 62 *Synechococcus*-like 16S rRNA alleles. Alleles falling within a 3%-diameter OTU centered on *Syn* OS-B′ are colored blue, the one allele within a 3%-diameter OTU centered on *Syn* OS-A is colored red, and other alleles are left unfilled. The gray region denotes the extent of the 3%-diameter OTU surrounding *Syn* OS-B′. The total number of reads at each distance is denoted by a dashed line. Percent divergence is shown on the upper x-axis.
(EPS)

S2 Fig Indel rates in coding and non-coding sequence. The probably of observing an indel relative to the most abundant allele at the same locus: in coding sequence (pink) and intergenic sequence (green) as a function of read position. The normalization is per read per site and only the deeply read loci are included. Frequency-weighted probabilities for the alleles inferred by *DADA* are shown as solid lines while probabilities for raw reads are shown by dashed lines. Alleles > 20% diverged from the most abundant were removed from these data due to concerns that misalignments may artificially produce indels. Probabilities were smoothed over a 25-bp window with a simple moving average. (Note that the indel frequencies for intergenic sites become noisy in the second half of the reads due to the small number of intergenic sites at those positions, and the opposite is true, although to a lesser extent, for the coding sites. This was due to primers being placed outside of the coding sequences that were being targeted.)
The indel probability shows stronger position dependence in coding sequence than in intergenic sequence, growing (roughly as a slow exponential of position) beyond 100nt and reaching ≈ 1% by 400nt in the raw reads, suggesting that sequencing error, which grows as a function of read position, is chiefly responsible for the coding sequence indels. After error correction, the whole curve is shifted down by nearly an order of magnitude, but the exponential is still evident, suggesting that what remains in coding sequence is likely to still be from errors. By contrast, in intergenic sequence there is little position-dependence either before or after error correction. Summing across all positions, the raw reads have an overall indel rate of 2.2 indels/400bp in coding sequence and 6.7 indels/400bp in intergenic regiosn. After denoising by *DADA*, these numbers were reduced to 0.51 an 5.3 respectively. Another distinction: a much larger fraction of the indels in intergenic sequence are not point indels; prior to error correction, 91% of coding sequence indels were length one while only 47% of intergenic indels were length one.
All these features of the indel data point to (1) the presence of a significant level of indel diversity in intergenic sequence, (2) the persistence of a low level of indel errors in coding sequence, and (3), assuming that the coding sequence indels are dominated by errors, an indel error rate of about 2/read (0.5% per site) with a slowly increasing rate along the reads.
(EPS)

S3 Fig The effect of false negatives on the pairwise distance spectrum. False negatives were drawn from the putative errors (see Methods) to conform to a liberal (0.30) and conservative (0.15) estimate of the expected *d*_*n*_/*d*_*s*_ ratio. The pairwise divergence spectrum for full alleles with no false negatives and with false negatives under each of the two *d*_*n*_/*d*_*s*_ ratios are shown for comparison with Fig 1. As can be seen, the effects of false negatives are negligible.
(EPS)

S4 FigSynonymous distance spectrum.The spectrum of pairwise synonymous distances *x* (magenta) and synonymous distances away from the most abundant allele (green) weighted by allele frequency. Only the 106 deeply read loci with a coding sequence ≥ 50 amino acids long are included. The spectrum for each locus was computed independently, and these spectra were then averaged together. The spectrum of distances from *Syn* OS-B′ to the most abundant allele at each locus is shown in black. A geometric distribution with $\bar{x}=4.5$ is shown in light purple.(EPS)

S5 Fig Distance spectrum away from *Syn* OS-B′. Magenta: the frequency-weighted spectrum of distances (excluding indels) between nucleotide alleles and *Syn* OS-B′ at deeply read loci. Green: the distance spectrum between the most abundant allele and the *Syn* OS-B′ allele across deeply read loci. Note that the absence of green points below 7 × 10^−3^ is due to limited number (n = 135) of loci. A geometric distribution with a mean of 8 is in light purple.
(EPS)

S6 Fig Distance spectrum away from *Syn* OS-A. The spectrum of distances (excluding indels) between nucleotide alleles and *Syn* OS-A at loci where *Syn* OS-A was ≥ 10% diverged from both *Syn* OS-B′ and the most abundant allele. To establish an *Syn* OS-A-like cloud, we kept only those alleles ≤ 5% diverged from *Syn* OS-A—the *Syn* OS-A “cloud”—and then kept only the 23 loci with 20+ reads in this cloud. The frequency-weighted spectrum of allele distances away from *Syn* OS-A are in magenta, and the spectrum away from the most abundant allele in this *Syn* OS-A cloud in green. In gray is the frequency-weighted pairwise distance spectrum between alleles within the *Syn* OS-A cloud. A geometric distribution with a mean of 0.75 is shown in light purple.
(EPS)

S7 Fig Outlier reads vs intergenic fraction of loci. The fraction of reads outside the main cloud (i.e. those > 10% diverged from the most abundant allele) as a function of the combined number of intergenic and hypothetical-protein sites across the deeply read loci. A least squares best fit is shown as a dashed black line, showing a very weak correlation that is robust to the cutoff used to define the main cloud.
(EPS)

S8 Fig Heterozygosity spectrum. The distribution of heterozygosity, *π*, for the main cloud alleles (orange) across the deeply read loci. In blue is the approximate *π* distribution under the null model of purely asexual drift with infinite sampling depth (using Theorem 1.28 in [82], keeping the first 300 terms in the sum and performing 10000 replicates). In green, we generated a sexual drift simulation with the number of loci and sampling depths matching the real data, and plot the distribution of *π* across these simulated loci. Although the observed distribution fits the asexual drift expectation relatively well, particularly in the large-*π* tail, there is a marked excess of loci with very low *π*—even more marked in comparison with the sexual simulations. These loci are notable for having one very high frequency allele. For example, of the 15 loci with *π* < 0.005, the average top allele frequency is 76%, more than twice the average top allele frequency across all loci (33%). The observed deviations from asexual neutral drift in the allele rank-frequency spectra (Fig 4) were predominantly due to this same subset of loci with reduced diversity and a higher-frequency-than-expected most-abundant allele. These low-heterozygosity loci also contribute to the excess at zero distance, relative to the exponential expectation, in the allele pairwise distance spectra of Fig 1.
(EPS)

S9 Fig *d*_*n*_/*d*_*s*_ from the metagenome. The spectrum of *d*_*n*_ and *d*_*s*_ for low-temperature OS metagenome reads relative to the *Syn* OS-B′ genome. 12194 metagenome reads with BLAST hits to *Syn* OS-B′ spanning their full length, and with alignments to *Syn* OS-B′ that involved no gaps, were used (see Methods). the gray line is *d*_*n*_/*d*_*s*_ = 0.125, the ratio of the maximum likelihood values of *d*_*n*_ and *d*_*s*_ for this collection of alignments. In blue, is shown a moving average of *d*_*n*_ vs *d*_*s*_, by dividing the reads into 30 bins in *d*_*s*_ with an equal number of reads in each, and then averaging *d*_*n*_ within each bin.
(EPS)

S10 FigLinkage decay for non-synonymous and downsampled-synonymous pairs of sites.The reciprocal of the average linkage correlations, 1/〈*r*^2^〉, as a function of the separation *x* of pairs of sites: amino acids (red), synonymous (green), and synonymous downsampled to match the non-synonymous frequency pair statistics (blue). The data were produced by first computing $r^{2}=\frac{{\left(f_{ab}-f_{a}f_{b}\right)}^{2}}{f_{A}f_{a}f_{B}f_{b}}$ for all pairs of sites, with *r*^2^ = 0 for pairs where one or both sites were fixed; *r*^2^ was then averaged over all pairs in each distance bin, and the inverse of this plotted. The left plot shows the full data with all the main cloud SNPs removed (i.e. only SNPs contributed by outlier alleles), while the right plot shows just the main cloud.(EPS)

S11 Fig Allele frequency correlations between low-temperature data sets. The probabilities (circles) for deep-alleles in different frequency bins to be found *exactly* (up to point indels) amongst the low-temperature OS metagenome reads. Only alleles in deeply read loci closer to *Syn* OS-B′ than *Syn* OS-A were considered (4576 in total). From the 543 OS metagenome reads, 184 (≈ 34% of the total) matched (up to indels) some allele in this restricted set. The black dashed line shows the expected fraction of alleles in different frequency bins to have matches amongst the OS reads under a model that 34% of the OS metagenome sample is identical with the population from the deep-amplicon-allele sample. Gray dashed lines give a 99% prediction interval. The probabilities for deep alleles in the different frequency bins to be identical with *Syn* OS-B′ alleles are shown by triangles.
(EPS)

S12 Fig Spectrum of the “*Syn* OS-A cloud” frequencies. At 117 of the deeply read loci, *Syn* OS-A was ≥ 10% diverged (including both substitutions and indels) from both *Syn* OS-B′ and the most abundant allele in pairwise alignments. At these loci, we recorded the cumulative frequency of all deep alleles ≤ 5% diverged from *Syn* OS-A—an *ad hoc* *Syn* OS-A-like cloud. The distribution of these allele frequencies is shown, along with a rough exponential fit with a mean of 0.7%.
(EPS)
