# Supplementary material for: Probing the ecological and evolutionary history of a thermophilic cyanobacterial population via statistical properties of its microdiversity
Source: PLoS One. 2018 Nov 14;13(11):e0205396. doi: 10.1371/journal.pone.0205396 (PMC6235289; doi:10.1371/journal.pone.0205396)
Supplement: S1 Table — TD = transition dimorph, D = dimorph. < 1% freq means that the minor variant of the polymorphism was at < 1%. “One allele” means the minor variant of the polymorphism was found in just one allele. “Alone” means the polymorphism was not found on an allele with any other < 1%, one allele polymorphisms. Within the main cloud, 75% of non-synonymous transition dimorphs are at < 1% frequency (compared with just 37% for the synonymous). Of these, 95% have their minor variant present in only one allele (compared with 83% for synonymous). Of this subset, 70% are alone (i.e. on alleles that contribute only one such SNP) compared with 56% for synonymous. No allele contributed > 3 < 1% frequency non-synonymous SNPs (compared with 16 for the synonymous). In the full data, a similar pattern was observed. 72% of non-synonymous transition dimorphs are at < 1% frequency, 87% of these are found in one allele, and 63% of these are alone. 38 alleles contribute > 3 < 1% frequency non-synonymous SNPs, and 9 is the maximum. So any linkage contributed by low-frequency outlier alleles does not overwhelm the features of the non-synonymous SNPs of the main cloud. Amino acid polymorphisms are similar: 71% of amino acid dimorphs in the main cloud have minor frequencies < 1%, of which 95% exist in only one allele, but (due to the inclusion of differences other than single second-site non-synonymous SNPs) only 47% of amino acid dimorphs are found alone, and the maximum number on a single allele is 13. In the full data, 70% of amino acid dimorphs are at < 1% frequency, 86% exist in only one allele, and 42% are alone. 35 alleles contribute > 6 < 1% frequency amino acid changes, and 23 is the maximum. (PDF) [file pone.0205396.s001.pdf]

SNP Type	Synonymous TDs	Non-synonymous TDs	Amino acid Ds
Main Cloud			
Total	2518	1029	2202
$< 1\%$ freq	936	772	1566
$< 1\%$ freq, one allele	777	732	1481
$< 1\%$ freq, one allele, alone	438	514	697
Full Data			
Total	4063	1490	3086
$< 1\%$ freq	1784	1077	2171
$< 1\%$ freq, one allele	1186	935	1861
$< 1\%$ freq, one allele, alone	408	589	775
